# Enhancement of Drug Seeking Following Drug Taking in a Sexual Context Requires Anterior Cingulate Cortex Activity in Male Rats

**DOI:** 10.3389/fnbeh.2020.00087

**Published:** 2020-06-25

**Authors:** Lindsey B. Kuiper, Kathryn A. Lucas, Vy Mai, Lique M. Coolen

**Affiliations:** ^1^Department of Neurobiology and Anatomical Sciences, University of Mississippi Medical Center, Jackson, MS, United States; ^2^Brain Health Research Institute, Kent State University, Kent, OH, United States; ^3^Department of Biological Sciences, Kent State University, Kent, OH, United States

**Keywords:** reinstatement, chemogenetics, anterior cingulate (ACC), sexual behavior, methamphetamine, relapse, addiction, prefrontal cortex

## Abstract

Individual variance in vulnerability to develop addictions is influenced by social factors. Specifically, drug-taking in a sexual context appears to enhance further drug-seeking behavior in human users, as these users identify the effects of drugs to enhance sexual pleasure as a primary reason for continued drug use. Methamphetamine (Meth) is commonly used in this context. Similarly, male rats that self-administered Meth immediately followed by sexual behavior display enhanced drug-seeking behavior, including attenuation of extinction and increased reinstatement to seeking of Meth-associated cues. Hence, drug-taking in a sexual context enhances vulnerability for addiction. However, the neural mechanisms by which this occurs are unknown. Here the hypothesis was tested that medial prefrontal cortex is essential for this effect of Meth and sex when experienced concurrently. First it was shown that CaMKII neurons in the anterior cingulate area (ACA) were co-activated by both Meth and sex. Next, chemogenetic inactivation of ACA CaMKII cells using AAV5-CaMKIIa-hM4Di-mCherry was shown not to affect Meth-induced locomotor activity or sexual behavior. Subsequently, chemogenetic inactivation of ACA CaMKII neurons during Meth self-administration followed by sexual behavior was shown to prevent the effects of Meth and sex on enhanced reinstatement of Meth-seeking but did not affect enhanced drug-seeking during extinction tests. These results indicate that ACA CaMKII cell activation during exposure to Meth in a sexual context plays an essential role in the subsequent enhancement of drug-seeking during reinstatement tests.

## Introduction

Substance use disorders are a severe threat to public health and influenced by social experiences (Heilig et al., 2016; Lacy et al., 2016; Robinson et al., 2016, 2017; de Wit and Sayette, 2018; Venniro et al., 2018, 2019). Drugs are often used in a social or sexual context, with commonly abused drugs including psychostimulants known to robustly increase sexual arousal and pleasure, whereas sexual enjoyment is diminished during drug abstinence (Lorvick et al., 2012; Safi et al., 2016; Kuiper and Coolen, 2018). Users identify increased sexual pleasure as a reason for drug use and relapse (Saw et al., 2018). Moreover, drug addiction is highly comorbid with compulsive sexual behavior, suggesting a shared neural basis for drug and non-drug addictions (Frohmader et al., 2010b; Tull et al., 2011; Ahmadi et al., 2017; Berry and Johnson, 2018; Kuiper and Coolen, 2018; Kuiper et al., 2019).

Previous studies utilized rat models to investigate increased vulnerability for drug addiction and hypersexuality in rats that were administered methamphetamine (Meth) immediately followed by sexual behavior. This has been termed concurrent Meth and sex in our previous publications (Frohmader et al., 2010c, 2011; Kuiper and Coolen, 2018; Kuiper et al., 2019), as sexual behavior is experienced while brain Meth levels are high (Riviere et al., 2000). It was demonstrated that concurrent Meth and sexual behavior leads to maladaptive sexual behavior persisiting for weeks (Frohmader et al., 2010a, 2011). Likewise, exposure to Meth in the sexual context increased drug-seeking behaviors in male rats measured by conditioned place preference (CPP) (Frohmader et al., 2011). Subsequent studies utilized operant drug self-administration paradigms in which male rats were restricted to a relatively low dose of Meth during limited numbers of daily sessions (Kuiper et al., 2019) in order to model the initial recreational use of human users (Cho et al., 2001). Following this limited Meth self-administration, animals were subsequently tested for drug-seeking behaviors in extinction sessions during which animals were placed back into the original Meth-taking environment but received no drug infusions or received no drug-associated cues nor infusions, and in reinstatement sessions, where drug-seeking was triggered by Meth-associated discrete cues, Meth administration, or by mating in the absence of Meth. Increased drug-seeking behaviors during extinction and reinstatement sessions are thought to parallel aspects of relapse to drug-taking in human drug users, which is a risk even after prolonged periods of drug abstinence (Bossert et al., 2013; Beloate and Coolen, 2017). As expected, male rats exposed to the restricted Meth self administration showed limited or no drug seeking behaviors during extinction sessions and during reinstatement testing. In contrast, male rats that experienced restricted Meth self-administration but each time followed immediately by sexual behavior (concurrent Meth and sex) showed enhanced drug-seeking behavior. Specifically, concurrent Meth and sex-treated animals responded significantly more on the drug-paired lever during extinction sessions and during reinstatement tests where drug-seeking was triggered by Meth-associated discrete cues, Meth administration, or by mating in the absence of Meth, compared to male rats exposed to non-concurrent Meth self-administration and sex (Kuiper et al., 2019).

The goal of the present set of studies is to examine the neural substrates mediating enhanced drug seeking behavior using this paradigm where Meth is taken in the sexual context. We hypothesize that the medial prefrontal cortex (mPFC) plays a critical role for this association between Meth and sexual behavior, as it plays a key role in learning and memory (Morici et al., 2015; Katzman and Alberini, 2018), including for drug-related cues (Peters et al., 2013; Riga et al., 2014; Stern et al., 2014). The homologous region in the human brain is implicated in compulsive disorders, including drug addiction and hypersexuality (Voon et al., 2014; Schmidt et al., 2017; Kuiper and Coolen, 2018) and male rats with mPFC lesions exhibit maladaptive sexual behavior (Davis et al., 2010). In addition, concurrent Meth and sex experience causes a long-lasting increase in basal neural activation, which was observed specifically in mPFC CaMKII- positive neurons, which putatively comprise the output projections from mPFC, and not in GABAergic neurons (Kuiper et al., 2017). Finally, mating and Meth co-activate neurons in the anterior cingulate area (ACA), but not in other subregions of the mPFC (Frohmader et al., 2010c). Therefore, the purpose of the current study was to test the hypothesis that activation of ACA CaMKII neurons during Meth taking while engaging in sexual behavior contributes to the subsequent enhancement of drug-seeking behavior. A commonly used cell-specific chemogenetic inactivation approach (DREADD; Urban and Roth, 2015; Campbell and Marchant, 2018) was used involving viral vector delivery of the inhibitory human muscarinic receptor hM4Di and activation of this designer receptor by Clozapine-*N*-oxide during operant drug self-administration approaches to examine drug-seeking behaviors during extinction and reinstatement sessions.

## Materials and Methods

### Animals

Male Sprague Dawley rats (226–250 g; Charles River Laboratories, Wilmington, MA) were pair-housed under a 12–12 h reversed light/dark cycle in a climate-controlled facility. Food and water were freely available. Females (Sprague Dawley, Charles River, 201–225 g) for mating partners were deeply anesthetized (isoflurane gas; Piramal, Bethlehem, PA), ovariectomized and implanted with subcutaneous silastic capsule (Dow Corning Corp., Midland, MI) containing 5% estradiol benzoate (Sigma-Aldrich, St. Louis, MO) and 95% cholesterol (Sigma-Aldrich). To induce sexual receptivity, females received progesterone (500 μg in 0.1 mL sesame oil, Sigma-Aldrich) 4 h prior to testing. All behavioral testing was conducted under dim red lights in the animals' dark phase. All animal procedures were approved by University of Mississippi Medical Center Institutional Animal Care and Use Committee and followed the guidelines outlined by the United States National Institutes of Health.

### Surgeries

#### Catheterization Surgery

Rats in self-administration experiments received chronic indwelling catheters (Instech; Plymouth Meeting, PA) into the right jugular vein under isoflurane gas anesthesia (2–3%) as described in detail in Kuiper et al. (2019). Animals were administered carprofen (5 mg/kg; s.c.) for analgesia prior to surgery and daily for 48 h post-operatively. Catheters were flushed with 0.1 mL gentamicin (5 mg/mL) and 0.1 mL heparinized saline (70 U/mL) daily for 1 week post-operatively. Thereafter, catheters were flushed with 0.1 mL heparinized saline before and after each self-administration session to confirm catheter patency.

#### Stereotaxic Surgery for DREADD Delivery

Rats received injections of AAV5-CaMKIIa-hM4Di-mCherry (University of North Carolina Vector Core, Chapel Hill, NC) 3 weeks before behavioral testing. Under deep anesthesia (2–3% isoflurane gas) in a stereotaxic frame (David Kopf Instruments, Tujunga, CA), microinjections (0.8–1 μL; 2 × 10^12^ vg/mL) using a 10 μL Hamilton syringe and 26-gauge microinjection needle (Hamilton Company; Reno, NV) were delivered bilaterally into the anterior cingulate area (ACA) at coordinates +2.9 AP, ± 0.6 ML from Bregma; −2.6 DV from top of skull in the DREADD validation experiment. The same coordinates were used in ACA DREADD self-administration experiment, but two injections were delivered to increase rostral-caudal spread throughout the ACA: +2.5 and +2.0 AP; ± 0.5 ML from Bregma; −3.0 DV from top of skull. For both ventral mPFC experiments, virus was injected at coordinates +3.0 A/P, ± 0.6 M/L from Bregma, −4.8 D/V from top of skull. Animals were given carprofen (5 mg/kg; s.c.) prior to surgery and once daily for the first 48 h after surgery.

### Drug Delivery

#### Methamphetamine

Methamphetamine hydrochloride (Sigma-Aldrich) was delivered either systemically (1 mg/ml/kg, in saline, s.c. or i.p.) or intravenously (via jugular vein catheters; 0.04 mg/kg per infusion in 0.166 mL). The dosages were selected to match our previous studies (Frohmader et al., 2010a,c, 2011; Kuiper et al., 2017, 2019).

#### Clozapine-N-Oxide Administration

Clozapine-*N*-oxide (CNO, Tocris Bioscience cat. # 4936, Avonmouth, Bristol, UK, 0.5, 1, or 3 mg/mL dissolved in 0.9% saline) was administered to activate hM4Di receptors, or vehicle (saline) was administered as control, s.c. 30 min prior to behavioral testing.

### Behavioral Testing

All analyses were conducted by experimenters blinded to experimental treatments.

#### Sexual Behavior

Mating behavior was tested in a separate mating arena (60 × 45 × 50 cm plexiglass cage containing clean bedding) with a receptive female until display of ejaculation and the first subsequent intromission. Sexual behavior parameters were recorded: mount latency (ML; time from introduction of female to the first mount), intromission latency (IL; time from introduction of female to first mount with vaginal penetration), ejaculation latency (EL; time from first intromission until ejaculation), post-ejaculation interval (PEI; time from ejaculation to next intromission), total mounts (M), total intromissions (IM), and total number of intromissions divided by total of mounts plus intromissions (copulation efficiency).

#### Meth Self-Administration and Operant Behavior

Self-administration and subsequent operant tests for behavior (extinction, reinstatement) were conducted based on our previously published publication (Kuiper et al., 2019). A standard two-lever operant chambers (30.5 × 24 × 21 cm; Med Associates, St. Albans, VT) with retractable levers and cue lights above each lever was used. Operant sessions started 2–3 h after onset of the dark phase, and each operant session lasted a maximum 3 h or until animals earned maximum (25) drug infusions. Meth-self-administration sessions were conducted using a fixed-ratio 1 (FR1) schedule of reinforcement such that each response on the active (left) lever resulted in an infusion of 0.04 mg/kg methamphetamine hydrochloride dissolved in sterile saline and a white light above the active lever was illuminated for 6 s; both levers retracted for 10 s after each active response. Responses on the inactive (right) lever were without programmed consequences. All lever responses (active and inactive) and numbers of infusions were recorded (Med Associates, MED-PC IV software, RRID:SCR_012156). During extinction and reinstatement, saline was used to fill the infusion lines to prevent negative pressure on the indwelling catheter; a saline syringe remained attached to the line but was not connected to the infusion pump.

#### Operant Extinction

Animals were subjected to 10 daily hour-long sessions during which lever responses had no programmed consequences.

#### Cue Extinction/Cue Reinstatement

Males were subjected to 1-h sessions during which each active lever response resulted in drug-paired cue presentation (cue light and pump sound). Responses on active and inactive levers were recorded.

#### Meth-Primed Reinstatement (Meth Reinstatement)

Males were injected with Meth (1 mg/kg, i.p.) 15 min prior to being subjected to a 1-h session during which responses on the active lever resulted in presentation of drug-paired cues (cue light and pump sound). Responses on active and inactive levers were recorded. Saline habituation injections (1 mL/kg, i.p.) were given 15 min. before the final extinction session preceding Meth Reinstatement and two daily cue extinction tests were conducted prior to this test.

#### Locomotor Activity

Animals were placed in Plexiglas open field activity chambers (Med Associates) equipped with 16 × 16 photobeam arrays. Horizontal ambulatory distance traveled in cm was analyzed using Med Associates Activity Monitor software and expressed as total distance (cm) per 15 min.

### Experimental Designs and Groups

#### Meth/Sex Induced Neural Activity Experiment (Experiments 1; Figure 1)

**Figure 1:**
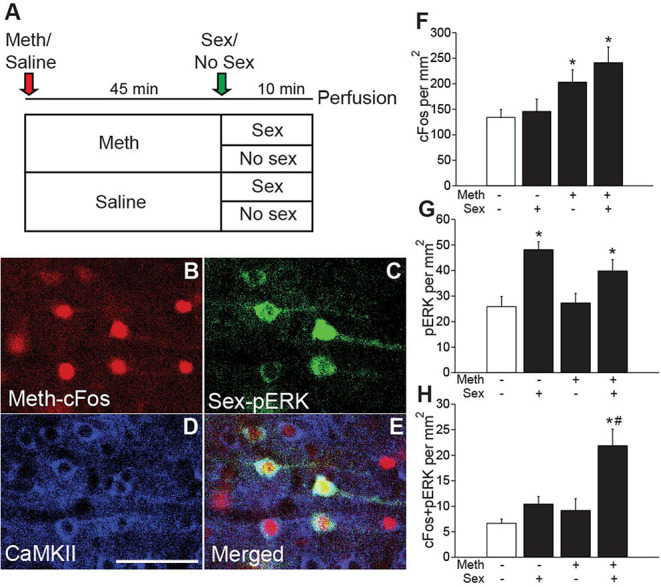
Meth-induced cFos and sex-induced pERK are co-expressed in CaMKII neurons. **(A)** Experimental design (Experiment 1). **(B)** Representative images of Meth-induced cFos, **(C)** sex-induced pERK, **(D)** CaMKII, and **(E)** merge in the ACA of a Meth/sex-treated animal. **(F)** cFos cell counts and **(G)** pERK cell counts for each group: Meth/Sex (*n* = 4), Meth/No Sex (*n* = 5), Saline/Sex (*n* = 5), and Saline/No Sex (*n* = 5). **(H)** Numbers of cells dual-labeled with cFos and pERK. * indicates significant increase vs. control, # indicates significant increase in double labeling vs. control or single treatments. All data are expressed as Mean ± SEM.

The goal of this study was to utilize the distinct temporal expression profiles of neuronal activity markers cFos (expression 30–90 min after stimulus) and pERK (expression 5–15 min after stimulus) to demonstrate co-activation by Meth and mating as described in our previous publication (Frohmader et al., 2010c). Males were placed in mating arena and were administered Meth (1 mg/kg; s.c.) or saline. Forty-five minutes later, males either mated with a receptive female or were left undisturbed. Thus, four groups were included in this study: Meth/Sex (*n* = 4), Meth/No Sex (*n* = 5), Saline/Sex (*n* = 5), and Saline/No Sex (*n* = 5). Ten minutes after introduction of female, and 55 min after injection of Meth, males were perfused to visualize Meth-induced cFos and sex-induced phosphorylation of MAP kinase (pERK). Experimental timeline shown in Figure 1A.

#### DREADD Validation Experiments (Experiments 2 and 4)

**Figure 2:**
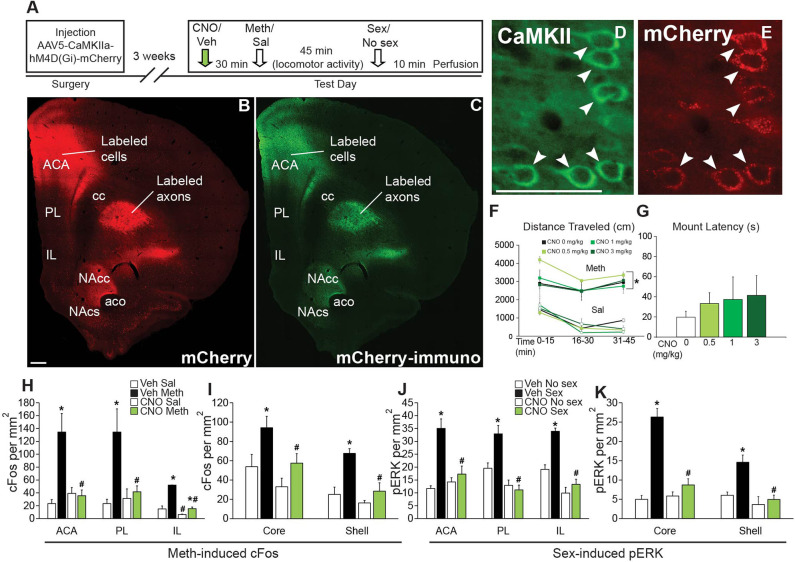
Chemogenetic inactivation of CaMKII neurons in the ACA does not affect Meth-induced locomotor activity or sexual behavior, but attenuates Meth-cFos and sex-pERK. **(A)** Experimental design(Experiment 2). **(B)** Representative scan of a section containing ACA endogenous mCherry and **(C)** immunofluorescence for mCherry. **(D)** Representative images of CaMKII immunofluorescence and coexpression of **(E)** mCherry. **(F)** Locomotor activity per 15 min for all CNO dosages. **(G)** Mounting latency for all CNO dosage groups. **(H)** cFos cell counts in the ACA, PL, and IL mFPC subregions. **(I)** cFos cell counts in the core and shell subregions of NAc. **(J)** pERK cell counts in the ACA, PL, and IL subregions of mPFC. **(K)** pERK cell counts in NAc core and shell. Experimental groups: CNO (1 mg/kg)/Meth/Sex (*n* = 4), CNO (1 mg/kg)/Sal/No Sex (*n* = 4), CNO (0.5 mg/kg)/Meth/Sex (*n* = 3), CNO (0.5 mg/kg)/Sal/No Sex (*n* = 3), CNO (3 mg/kg)/Meth/Sex (*n* = 3), CNO (3 mg/kg)/Sal/No Sex (*n* = 3), Veh/Meth/Sex (*n* = 4), and Veh/Sal/No Sex (*n* = 4). ACA, anterior cingulate area; PL, prelimbic cortex; IL, infralimbic cortex; mPFC, medial prefrontal cortex; NAc, nucleus accumbens. * indicates effects of Meth on locomotor activity, cFos- and pERK- immunoreactivity; # indicates significant reduction of cFos- or pERK-immunoreactivity by CNO. All data are expressed as Mean ± SEM.

The main objective of these experiments was to confirm CAMKII cell-specific expression of hM4Di-mCherry and lack of effects of CNO on baseline locomotor and mating activity. Animals received stereotaxic injections of AAV5-CaMKIIa-hM4Di-mCherry into the anterior cingulate area (ACA; Experiment 2; Experimental timeline shown in Figure 2A) or vmPFC (Experiment 4; Experimental timeline shown in **Figure 4A**) and received sexual experience (4 ×) during the 3 weeks after viral transduction. In addition, animals were injected with saline (1 mL/kg s.c.) and measured for baseline locomotor activity in the 3 days prior to the final test for habituation to testing conditions. During the final test, animals received either vehicle (saline) or one of three doses of CNO (the commonly used dose of 1 ml/kg, and lower or higher dosages of 0.5 or 3 mg/kg, s.c.) 30 min prior to an injection with Meth (1 mg/kg; s.c., i.e., passive administration) or saline. Locomotor activity was measured for 45 min. Next, animals that received Meth mated with a receptive female, while males that received vehicle were left undisturbed. Ten min after introduction of female or equivalent time, rats were perfused for analysis of Meth-induced cFos and sex-induced pERK. The following groups were included for behavioral analysis in the ACA DREADD experiment (Figure 2): CNO (1 mg/kg)/Meth/Sex (*n* = 4), CNO (1 mg/kg)/Sal/No Sex (*n* = 4), CNO (0.5 mg/kg)/Meth/Sex (*n* = 3), CNO (0.5 mg/kg)/Sal/No Sex (*n* = 3), CNO (3 mg/kg)/Meth/Sex (*n* = 3), CNO (3 mg/kg)/Sal/No Sex (*n* = 3), Veh/Meth/Sex (*n* = 4), and Veh/Sal/No Sex (*n* = 4). The following groups were included for behavioral analysis in the vmPFC DREADD experiment (Figure 4): CNO (1 mg/kg)/Meth/sex (*n* = 3), CNO (1 mg/kg)/Sal/No Sex (*n* = 3), Veh/Meth/Sex (*n* = 3), and Veh/Sal/No Sex (*n* = 3). For cFos/pERK analysis, only the 1 mg/kg CNO and corresponding control groups were included. DREADD verification was conducted on all animals.

#### DREADD Meth/Sex Self-Administration Experiments (Experiments 3 and 5)

**Figure 3:**
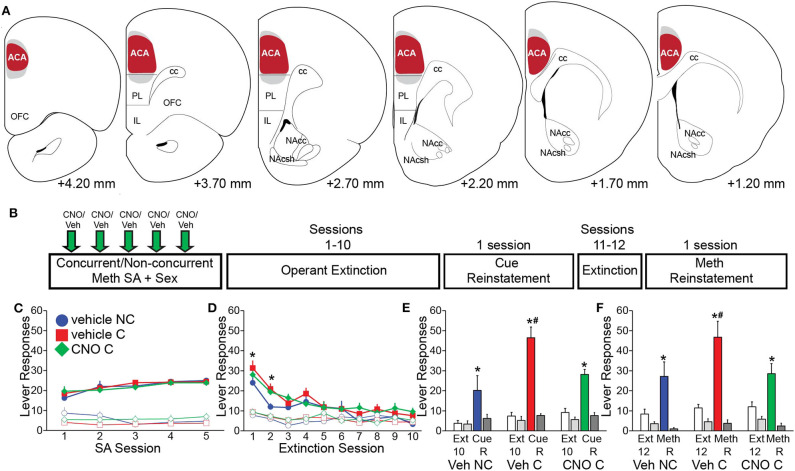
Chemogenetic inactivation of CaMKII neurons in the ACA attenuates drug-seeking induced by concurrent Meth self-administration and sex. **(A)** Diagrams of virus spread through the ACA (Experiment 3). 97.3% mCherry-positive cells co-localized CaMKII; 87.5% CaMKII-positive cells co-localized mCherry (red shading: area of mCherry expression in all animals; gray shading: 5 out of 37 animals with mCherry expression slightly outside of ACA). **(B)** Experimental design for **(C–F)** (Experiment 3). **(C)** Active (closed markers) inactive (open markers) lever responses for vehicle non-concurrent (*n* = 9, blue circles), vehicle concurrent (*n* = 14, red squares), and CNO concurrent (*n* = 14, green diamonds) Meth self-administration. **(D)** Active and inactive lever responses during 10 operant extinction sessions (*indicates significant difference from vehicle non-concurrent). **(E)** Active (solid white and solid color) and inactive (solid light and dark gray) lever responses during Cue Reinstatement (vs. the previous 3 extinction sessions) and during **(F)** Meth Reinstatement (vs. previous 2 extinction sessions). C, concurrent; NC, non-concurrent; CNO, clozapine-*N*-oxide. # indicates significantly increased lever responding in concurrent animals vs. non-concurrent and * indicates significant reinstatement vs. extinction in panels e and f. All data are expressed as Mean ± SEM.

**Figure 4:**
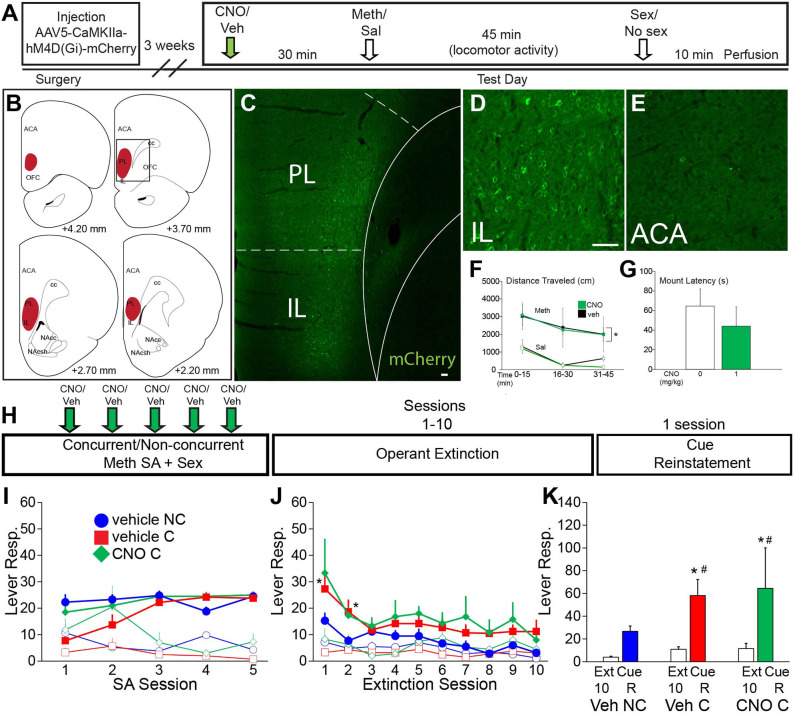
VMPFC activity is not essential for the effects of concurrent Meth self-administration and sex on drug seeking behavior. **(A)** Experiment timeline for B-G (Experiment 4). **(B)** Diagrams of DREADD virus spread through the vmPFC. **(C)** Representative scan of a section containing vmPFC immunofluorescence for mCherry. **(D)** High magnification of mCherry-positive immunolabeling in IL. **(E)** High magnification of lack of mCherry-positive immunolabeling in ACA. **(F)** Locomotor activity in 15 min time blocks for animals treated with 1 mg/kg CNO or vehicle (*n* = 3 per group; closed squares, Meth treated; open squares, saline control). CNO did not affect Meth-induced locomotor activity [Meth vs. sal, *F*_(3,21)_ = 8.79, *p* < 0.001] (*indicates significant difference from saline). **(G)** Latency to initiate mating behavior for all CNO dosage groups. CNO did not affect mating behavior. **(H)** Experimental timeline for I-K (Experiment 5). Groups: vehicle non-concurrent (*n* = 6), vehicle concurrent (*n* = 6) and CNO concurrent (*n* = 4). **(I)** Operant behavior during acquisition of self-administration active (closed markers) inactive (open markers) lever responses for vehicle non-concurrent (blue circles), vehicle concurrent (red squares), and CNO concurrent (green diamonds). Levers were retracted upon infusion of drug for 10 s. **(J)** Active and inactive lever responses during 10 operant extinction sessions (*indicates significant difference from vehicle non-concurrent). **(K)** Active lever responses during Cue Reinstatement, compared with the average of the previous 3 extinction sessions. Scale bars = 50 μm. All data are expressed as Mean ± SEM (*indicates significant difference from responding during extinction (Ext). # indicates difference from vehicle non-concurrent).

The goal of these experiments was to test effects of CAMKII cell inhibition during Meth self-administration and sexual behavior on subsequent drug seeking behaviors. Animals received injections of AAV5-CaMKIIa-hM4Di-mCherry into the ACA (Experiment 3; Experimental timeline shown in Figure 3A) or ventral mPFC (Experiment 5; Figure 4H). In Experiment 3 (ACA DREADD), non-concurrent animals received sexual experience during 5 daily sessions prior to Meth self-administration, while concurrent animals' sexual experience took place immediately after each Meth self-administration session. Animals first received two sessions of Meth self-administration prior to the start of the study (in an attempt to minimize overall session duration during CNO treatments; these prior sessions did not result in differences in Meth intake between groups, see Table S4). Animals were then either treated with CNO (1 mg/kg, s.c.) or vehicle (saline), 30 min prior to each Meth self-administration session. Thus, groups were: vehicle non-concurrent (*n* = 9), vehicle concurrent (*n* = 14), and CNO concurrent (*n* = 14). The average duration of the 5 Meth self-administration sessions did not differ between groups: 103 ± 6 min vehicle concurrent group, 114 ± 7 min CNO concurrent group, and 104 ± 9 vehicle non-concurrent group. Groups did not differ in any mating parameter. The average durations of the mating sessions after Meth self-administration were 8.3 ± 0.4 min vehicle group and 11.1 ± 0.9 min CNO group. The 5 Meth self-administration sessions were followed by 10 days of operant extinction and a cue reinstatement test, two additional cue extinction sessions and a Meth-primed reinstatement test. No mating took place during the extinction and reinstatement phases. One week after the last session, males were perfused for verification of DREADD expression. Prior to perfusion, all groups received CNO (1 mg/kg; s.c.), followed 30 min later by Meth (1 mg/kg; s.c.) and sexual behavior at 50 min after Meth (for 10 min), immediately followed by perfusion to visualize effects of CNO on Meth-induced cFos and mating-induced pERK.

Experiment 5 (vmPFC DREADD) was conducted using the same methods as the ACA DREADD self-administration experiment (Experimental timeline shown in Figure 4H) and including the following groups: vehicle non-concurrent (*n* = 6), vehicle concurrent (*n* = 6), and CNO concurrent (*n* = 4). The average duration of the 5 Meth self-administration sessions did not differ between groups (132 ± 11 min vehicle concurrent group, 119 ± 11 min CNO concurrent group, and 131 ± 9 min vehicle non-concurrent group). The average duration of the mating sessions after Meth self-administration: 10.9 + 1.1 vehicle group and 10.1 + 2.0 CNO group.

### Criteria for Behavioral Experiments

#### Criteria for Self-Administration and Exclusion

Criteria for stable drug administration was set to at least 3 consecutive days/sessions earning 23–25 infusions/session and significant differences between active and inactive lever responding. In addition, if animals in the concurrent groups in the initial self-administration sessions received two or fewer drug infusions, they did not receive subsequent concurrent mating sessions. This occurred in only one or two sessions and in only a small subset of animals (4 out of 38 animals, or 10.5%, distributed equally among groups). Animals that lost catheter patency (verified by ease of flushing and preference for the active lever) during the self-administration sessions were excluded from all data analysis.

#### Criteria for Mating and Exclusion

Mating criteria were not pre-determined for non-concurrent or concurrent males. All animals displayed mating behavior (except for 1 animal) and behavioral parameters are shown for all experiments in Table S5. Numbers of mounts, intromissions, and ejaculations were recorded, as well as latencies to first mount, intromission, ejaculation and post-ejaculatory interval (defined as time from ejaculation to subsequent intromission). Data for each parameter was included in analysis only for animals that displayed the parameter.

#### Criteria for Operant or Cue Extinction and Exclusion

Since concurrent and non-concurrent groups differed in active lever pressing on the first day, criteria for extinction were not set as a percent change from the first day of extinction. Instead, extinction training was conducted until group differences in active lever presses were not detected for two or more consecutive sessions. Animals were excluded for high inactive lever pressing that was >2 standard deviations from the group average (2 veh non-concurrent animals).

### Brain Collection and Sectioning

Rats were deeply anesthetized using sodium pentobarbital (270 mg/kg, i.p.) and transcardially perfused using 10 mL of 0.9% saline followed by 500 mL of 4% paraformaldehyde in 0.1 M phosphate buffer (PB). Brains were removed, post-fixed in paraformaldehyde for 1 h at room temperature, and stored in 20% sucrose dissolved in PB containing 0.01% sodium azide at 4°C. Coronal sections (35 μm) were sectioned into 4 parallel series on a freezing microtome (Leica Biosystems SM2000R, Wetzlar, Germany) and stored at 20°C in 30% sucrose with 30% ethylene glycol, and 1% polyvinylpyrrolidone in PB.

### Immunohistochemistry

All incubations were performed with gentle agitation at room temperature. Free-floating sections were washed thoroughly with phosphate-buffered saline (PBS) before and between incubations. Sections were incubated in 1% H_2_O_2_ for 10 min, washed in PBS, and then blocked in incubation solution containing 0.1% bovine serum albumin and 0.4% Triton X-100 in PBS for 1 h prior to 17-h incubations with each primary antibody (diluted in the incubation solution). After staining, sections were mounted on SuperFrost Plus glass slides and coverslipped with an aqueous mounting medium (Gelvatol) containing the anti-fading agent 1,4-diazabicyclo(2,2)octane (DABCO; 50 mg/mL, Sigma-Aldrich) and stored in the dark at 4°C (fluorescence) or were dehydrated and coverslipped with dibutyl phthalate xylene mounting medium (Electron Microscopy Sciences, Hatfield, PA) (chromogen-DAB). Sections of all experimental groups within experiments were immunoprocessed simultaneously.

#### Immunohistochemistry Reagents

Extensive details regarding all antibodies and reagents used for immunohistochemistry are summarized in Table 1.

**Table 1:** Detailed information for all antibodies and reagents used for immunohistochemistry.

**Target/Host**	**Concentration**	**Company**	**RRID**
**PRIMARY ANTIBODIES**			
pERK (p44/42 mitogen activated protein kinase)/rabbit	1:4,000	Cell Signaling cat. # 9101	AB_331646
cFos/rabbit	1:100 immunofluorescence,!!!break!!! 1:4,000 immunoperoxidase	Santa Cruz prod. # sc-52	AB_2106783
CaMKII/mouse	1:1,000	Thermo Fisher Scientific cat. #MA1-048	AB_325403
mCherry	1:4,000	Abcam prod. # ab167453	AB_2571870
**SECONDARY ANTIBODIES**			
**Name**			
Biotinylated goat anti-rabbit	1:500	Vector Laboratories cat. # BA-1000	AB_2313606
Alexa-Fluor 555-conjugated goat anti-rabbit	1:100	Molecular Probes cat. # A21428	AB_141784
Cy5 conjugated donkey anti-mouse	1:100	Jackson Immunoresearch Laboratories cat. # 715-175-150	AB_2340819
Dylight 488 goat anti-rabbit	1:100	Thermo Fisher Scientific prod. # 35553	AB_1965947
**OTHER REAGENTS**			
**Name**			
ABC-elite	1:1,000 in PBS	Vecor Laboratories cat. #PK-6100	AB_2336819
Biotinylated tyramine tissue sample amplification (TSA)	1:250 (in PBS containing μL/mL of 3% H_2_O_2)_	PerkinElmer Life Sciences prod. # NEL700/700A	–
Dylight 488 conjugated streptavidin	1:100 in PBS	Thermo Fisher Scientific prod. # 21832	–
DAB (0.02% 3-3′ Diaminobenzidine tetrahydrochloride)	10 mg per 50 mL PB with 0.015% H_2_O_2_; enhanced with 0.02 nickel sulfate	Sigma-Aldrich prod. # D5905	–

#### cFos/pERK/CaMKII Triple Fluorescence

Sections containing frontal cortex from males receiving Meth or saline and sex or control and from animals receiving DREADD and passive Meth and sex were processed to visualize Meth-induced cFos and sex-induced pERK. Sections were incubated with rabbit antibody specific for p44/42 mitogen activated protein kinase (pERK, 1:4,000; overnight; Cell Signaling cat. # 9101, Danvers, MA; RRID:AB_331646), biotinylated goat anti-rabbit antibody (1:500; 1 h; Vector Laboratories cat. # BA-1000, Burlingame, CA; RRID:AB_2313606), avidin horseradish peroxidase complex (ABC-elite, 1:1,000; 1 hr; Vector Laboratories cat. # PK-6100; RRID:AB_2336819), biotinylated tyramine tissue sample amplification (TSA, 1:250 in PBS containing 1 μL/mL of 3% H_2_O_2;_ 10 min; PerkinElmer Life Sciences prod. # NEL700/700A, Boston, MA), and Dylight 488 conjugated streptavidin (1:100 in PBS; 30 min; Thermo Fisher Scientific prod. # 21832; Waltham, MA). Next, sections were incubated overnight in rabbit c-Fos antibody (1:100; Santa Cruz Biotechnology prod. # sc-52, Dallas, TX; RRID:AB_2106783) and mouse CaMKII antibody (1:1,000; Thermo Fisher Scientific cat. #MA1-048; RRID:AB_325403), Alexa-Fluor 555-conjugated goat anti-rabbit (1:100; 30 min; Molecular Probes cat. # A21428, Eugene, OR; RRID:AB_141784), and Cy5 conjugated donkey anti-mouse (1:00; 30 min; Jackson Immunoresearch Laboratories cat. # 715-175-150; West Grove, PA, USA; RRID:AB_2340819). All antibodies and the triple label protocol have been extensively validated in rat tissue (Frohmader et al., 2010c; Fanous et al., 2013; Gholizadeh et al., 2013) and omission of either primary antibody resulted in complete lack of staining in the corresponding wavelength.

#### mCherry/CaMKII Dual Fluorescence

To verify that DREADD expression was limited to ACA CaMKII-expressing neurons, sections containing frontal cortex and striatum from all animals receiving DREADD were immunostained for mCherry and CaMKII and sections throughout the brain stained for mCherry. Sections were incubated overnight with a rabbit antibody specific for mCherry (1:4,000; Abcam prod. # ab167453, Cambridge, MA; RRID:AB_2571870) and mouse CaMKII antibody (1:1,000; Thermo Fisher Scientific), followed by incubation with Dylight 488 goat anti-rabbit antibody (1:100; 30 min; Thermo Fisher Scientific prod. # 35553; RRID:AB_1965947) and Cy5-conjugated donkey anti-mouse antibody (1:100; 30 min; Jackson Immunoresearch Laboratories). mCherry immunostaining in cells was exclusively co-localized with endogenous red fluorescence indicating mCherry, as previously shown (Beloate et al., 2016) and further validating the antibody. CaMKII antibody has previously been validated (Fanous et al., 2013; Kuiper et al., 2017).

#### c-Fos Peroxidase Immunostaining

To analyze c-Fos induced by Meth, sections of mPFC, striatum and hypothalamus from all animals receiving DREADD and passive Meth and sex were stained for c-Fos and pERK using peroxidase-based chromogen staining. Sections were incubated overnight in c-Fos antibody (1:4,000; Santa Cruz) and then in biotinylated goat anti-rabbit antibody (1:500; 1 h; Vector Laboratories), ABC-elite (1:1,000; 1 h; Vector Laboratories), and a chromogen solution containing 0.02% 3-3′ Diaminobenzidine tetrahydrochloride (DAB; Sigma-Aldrich prod. # D5905; Lennette, 1978) enhanced with 0.02% nickel sulfate, with 0.015% H_2_O_2_ in PB (10 min).

#### pERK Peroxidase Immunostaining

To analyze pERK induced by sex, sections containing mPFC and striatum from all animals receiving DREADD and passive Meth and sex were incubated overnight in pERK antibody (1:4,000; Cell Signaling) and then in biotinylated goat anti-rabbit antibody (1:500; 1 h; Vector Laboratories), ABC-elite (1:1,000, 1 h; Vector Laboratories), and 0.02% DAB (Sigma-Aldrich) with 0.015% H_2_O_2_ in PB (10 min).

### Quantitative Analysis of Immunohistochemistry

#### Cell Counts

Images for analysis of fluorescently-labeled cells in the cFos/pERK experiment were captured at 20 × magnification using a CCD camera (Leica FX340) attached to a Leica microscope (Leica DM500) using the Leica Application Suite software (RRID:SCR_013673) and at 2 different focus planes on the z-axis. Single-labeled cFos-positive and pERK-positive cells and double-labeled cells were counted on these images. In all DREADD experiments, sections throughout the entire brain were examined for the presence of mCherry immunolabeling. In the DREADD experiments, images were captured using a cooled CCD camera (Microfire, Optronics, Goleta, CA, USA) attached to a Leica microscope (Leica DM500B) and Neurolucida software (MicroBrightField Bioscience, Williston, VT; RRID:SCR_001775). Cells were also counted using Neurolucida software. Single and double-labeled cells for mCherry and CaMKII for the DREADD Meth/sex passive administration and DREADD self-administration experiments were counted at the injection site (ACA 500 × 500 μm) on images taken at 40 × magnification. Single-labeled cells for cFos and pERK for this experiment were counted in the ACA, PL, and IL of the mPFC and in the core and shell subregions of the nucleus accumbens with a standard area of analysis using a light microscope and a drawing tube attachment at 10 × magnification (Leica DMR). Fluorescent single, dual, and triple-labeled cells for cFos, pERK, and CaMKII were counted in the ACA for both the Meth/sex passive administration experiment and in the DREADD self-administration experiment on virtual scans taken at 10 × magnification Standard areas of analysis in the mPFC were 1,200 × 800 μm for the ACA, 1,000 × 800 μm for the PL and IL, and 400 × 600 μm in the NAc shell and core for light microscopy, and 600 × 800 μm in the ACA for fluorescence microscopy. All analyses were conducted by experimenters blinded to experimental treatments.

### Statistical Analyses

Statistical analyses were performed using SigmaPlot software (Systat Software, Inc., San Jose, CA; RRID:SCR_003210). Detailed information for all statistical analyses can be found in Tables S1, S2, S6, S8. Normality and equal distribution were confirmed for all data.

#### Meth Self-Administration

Active lever responses, inactive lever responses, infusions earned, and mating parameters during repeated mating sessions were compared between and within groups using two-way repeated measures ANOVA with Holm-Sidak *post-hoc* pairwise comparisons. Active and inactive lever responses during reinstatement were compared using two-way ANOVA with the Holm-Sidak method for *post-hoc* pairwise comparisons. The self-administration experiment was conducted in 2 cohorts. Each cohort included all experimental groups and all experimental effects (of concurrent Meth/sex or DREADD) were replicated within each cohort. No statistical differences were detected between cohorts within groups, and data from the separate cohorts were therefore combined for the final numbers of animals.

#### Sexual Behavior

Parameters for sexual behavior were compared using Student's *t*-tests or one-way ANOVA.

#### Cell Counts

Numbers of cFos and pERK cells in the Meth/sex passive administration and the DREADD Meth/sex passive administration experiment were compared using two-way ANOVA with the Holm-Sidak method for *post-hoc* pairwise comparisons.

## Results

### Identification of Anterior Cingulate Cortex CaMKII Neurons Involved in Concurrent Meth and Sex Association

We previously demonstrated that Meth and mating co-activate neurons in the ACA ultilizing the distinct temporal expression profiles of neuronal activity markers cFos (expression 30–90 min after stimulus) and pERK (expression 5–15 min after stimulus) (Frohmader et al., 2010c). The goal of the first study (Experiment 1) was to provide the neurochemical identification of this co-activated ACA neuronal population (experimental timeline shown in Figure 1A; see Table S1 for details of statistical analyses). Male rats received either Meth or saline administration (passive administration) and half of the groups mated with a receptive female. No statistically significant differences in sexual behavior were detected between Meth- and saline- treated groups confirming previous reports (Frohmader et al., 2010c). Meth significantly increased numbers of cFos-ir neurons compared to saline [*F*_(1,15)_ = 12.1, *p* = 0.003; Figure 1F], and sexual behavior significantly increased numbers of pERK-ir cells compared to control [*F*_(1,15)_ = 20.5, *p* < 0.001; Figure 1G]. Moreover, Meth and sex co-activated significantly more neurons than groups treated with saline/sex [*F*_(1,15)_ = 11.9, *p* = 0.004; Figure 1H] or Meth/no sex [*F*_(1,15)_ = 16.6, *p* = 0.001; Figure 1H], and a significant interaction of factors Meth and sex [*F*_(1,15)_ = 4.9, *p* = 0.043] was detected. Replicating our previous findings (Frohmader et al., 2010c), 54.9% of mating-induced pERK neurons co-expressed Meth-induced cFos. Moreover, CaMKII was detected in 91.4% of co-activated neurons, 88.2% of single Meth-cFos neurons, and 95.5% of single sex-pERK neurons in animals co-treated with Meth and sex (Figures 1D,E). Hence, we hypothesize that ACA CaMKII neurons are positioned to contribute to the effects of concurrent Meth and sex on subsequent drug-seeking behavior. This hypothesis was tested using chemogenetic inactivation of ACA CAMKII neurons.

### Effects of Chemogenetic Inactivation of ACA on Mating and Meth-Induced Behavior and Neuronal Activation

First, a small validation experiment (Experiment 2; experimental timeline shown in Figure 2A) was conducted to confirm the specificity of the DREADD transduction and effects of CAMKII neuron inhibition on neuronal activation after Meth administration (passive) and mating behavior. mCherry-immunoreactivity was observed to be confined to CaMKII-expressing neurons in the ACA (91.9% of CaMKII-positive neurons expressed mCherry; 91.6% of mCherry-positive neurons expressed CaMKII; Figures 2B–E). mCherry immunoreactive cells were not present in the ventral mPFC, except for a limited spread to the dorsal prelimbic area (PL) in only a small portion of animals (2 out of 16 or 12.5%). Moreover, mCherry-immunoreactivity was absent from non-CaMKII cells in ACA and there was no immunolabeling of mCherry in other brain areas, demonstrating lack of retrograde transport of the virus. The presence of mCherry-immunoreactive axons was noted in areas known to receive inputs from the ACA (Balfour et al., 2006; Riga et al., 2014), including the caudate putamen, and nucleus accumbens (NAc) core and shell (Figures 2B,C), with axon projections gradually appearing less dense from dorsal to ventromedial consistent with previous reports (Gabbott et al., 2005; Balfour et al., 2006). CNO had no effect on baseline locomotor activity following saline injections at any dose tested, including the commonly used dose of 1 ml/kg, and lower or higher dosages (0.5 or 3 mg/kg) (Figure 2F; see Table S2 for all statistical analyses). CNO also did not disrupt Meth-induced hyperlocomotion [*F*_(7,40)_ = 17.7, *p* < 0.001; Meth vs. saline within all doses of CNO *p* = 0.002 – < 0.001; Figure 2F] or the ability to initiate mating behavior (Figure 2G). However, CNO (1 mg/kg dose) did prevent activation of ACA neurons as CNO attenuated Meth-induced cFos [*F*_(1,10)_ = 8.6, *p* = 0.015] and sex-induced pERK [*F*_(1,9)_ = 9.1, *p* = 0.015] in ACA (Figures 2H,J), whereas CNO treatment did not affect baseline cFos and pERK levels in non-mating saline-treated control animals (Figures 2H,J). In addition, Meth-cFos and sex-pERK were attenuated in NAc core [cFos: *F*_(1,10)_ = 6.4, *p* = 0.030; pERK: *F*_(1,9)_ = 31.9, *p* < 0.001] and shell [cFos: *F*_(1,10)_ = 11.2, *p* = 0.007; pERK: *F*_(1,9)_ = 16.7, *p* = 0.003] of CNO-treated animals (Figures 2I,K), suggesting that activation of NAc is partially dependent upon ACA inputs and consistent with a previous report that mating-induced cFos is expressed in NAc neurons that primarily receive axon inputs from ACA (Balfour et al., 2006). Despite a complete lack of DREADD expression (i.e., mCherry-immunolabeling) in cell bodies in the ventral regions of the mPFC, CNO also attenuated neural activation in prelimbic [PL; cFos: *F*_(1,10)_ = 5.9, *p* = 0.036; pERK: *F*_(1,9)_ = 33.2, *p* < 0.001] and infralimbic [IL; cFos: *F*_(1,10)_ = 51.9, *p* < 0.001; pERK: *F*_(1,9)_ = 60.6, *p* < 0.001] subregions of the mPFC (Figures 2H,J). These findings suggest that ACA inputs to these areas contribute to neural activation, consistent with previous reports of anatomical connections between the ACA and other mPFC subregions (Balfour et al., 2006; Hoover and Vertes, 2007). Indeed, some mCherry immunoreactive axon terminals were noted in these mPFC subregions (not visible in the low magnification used for images in in Figure 2). Finally, CNO did not affect Meth-induced cFos in the ventral pallidum, or sex-induced pERK in the medial preoptic area, areas known to be activated by Meth or sex behavior, respectively. These areas do not receive major ACA inputs (Table S3), indicating that effects of CNO were restricted to ACA CaMKII cells and their direct efferent targets. Together, these findings confirmed the use of inhibitory DREADDs in ACA CaMKII neurons to inhibit neural activity in CaMKII cells in ACA and their neural targets during Meth and sex experience without affecting initiation of mating- or Meth-induced behavioral responses. This study formed the basis for subsequently testing whether ACA CaMKII neurons and their targets contribute to the effects of concurrent Meth and sex on subsequent Meth seeking behavior.

### Chemogenetic Inactivation of ACA Prevents Effects of Concurrent Meth/Sex Treatment on Drug-Seeking During Reinstatement

Next, we tested whether preventing the activation of ACA CaMKII neurons during concurrent Meth and sex experience in the operant self administration paradigm would prevent subsequent effects on Meth-seeking behavior (Experiment 3; experimental timeline shown in Figure 3B). Chemogenetic inhibition of ACA CaMKII cells did not affect Meth taking behavior and groups did not differ in numbers of active responses, infusions earned, inactive responses, and total drug intake (Figure 3C, Table S4). There were also no differences between groups in sexual behavior (Table S5). CNO was not administered during the remainder of the experiment (Figure 3B), hence any differences between the CNO and vehicle concurrent groups were the result of inhibition of ACA CaMKII cells during the concurrent Meth and sex exposure and were not caused by differences in prior Meth taking or sex experience.

Replicating previous findings (Kuiper et al., 2019), the vehicle-treated concurrent group responded more on the active lever than the vehicle non-concurrent group during session 1 of extinction training [*t*_(14)_ = 1.7, *p* = 0.045] and both concurrent groups responded more on the active lever than vehicle non-concurrent males during session 2 of extinction training [*t*_(14)_ = 1.7, *p* = 0.020–0.025]. However, prior CNO treatment had no effect on this enhanced drug-seeking behavior as vehicle and CNO-pretreated concurrent males did not differ (Figure 3D; see Table S6 for all statistical analyses).

During reinstatement tests, all groups significantly increased responding during cue reinstatement compared to the last day of extinction [*F*_(1,46)_ = 64.1, *p* < 0.001; Figure 3E]. The vehicle concurrent group responded significantly more on the active lever than the vehicle non-concurrent group during the reinstatement test [*F*_(2,46)_ = 7.3, *p* = 0.002, two-way ANOVA, pairwise comparison veh concurrent vs. veh non-concurrent within Cue Reinstatement *p* < 0.001; Figure 3E], replicating our findings that concurrent Meth/sex enhanced reinstatement (Kuiper et al., 2019). However, the enhancement of reinstatement of Meth-seeking after concurrent Meth/sex was prevented by prior inhibition of CaMKII ACA cells (Figure 3E). The CNO-pretreated concurrent animals responded significantly less on the active lever compared to the concurrent vehicle-treated group [*F*_(2,46)_ = 7.3, *p* = 0.002; veh C vs. CNO C within Cue Reinstatement *p* < 0.001; Figure 3E] and did not differ from the non-concurrent vehicle control group (Figure 3E). Analysis revealed a group by session interaction during Cue Reinstatement [*F*_(2,46)_ = 6.1, *p* = 0.004]. Similar effects of CNO pretreatment were noted for Meth-primed reinstatement (Figure 3F). Meth-induced reinstatement of active responding was observed in all groups [*F*_(1,44)_ = 29.1, *p* < 0.001; Figure 3F]. As before, vehicle concurrent males responded more on the active lever compared to non-concurrent males, and although the main factor comparison failed to reach significance in the ANOVA [*F*_(2,44)_ = 2.6, *p* = 0.086], single pairwise comparison revealed significant effects [*t*_(12)_ = 2.4, *p* = 0.011; Figure 3F]. In contrast, the CNO-pretreated concurrent group responded significantly less than vehicle concurrent males [*t*_(12)_ = 2.8, *p* = 0.004; Figure 3F] and did not differ from non-concurrent controls.

One week after the final reinstatement test, CNO-induced reduction of neural activity induced by Meth (1 ml/kg, passive administration) and mating behavior, and specificity of DREADD (mCherry) expression was confirmed. 88% of ACA CaMKII cells co-localized mCherry immunoreactivity and mCherry expression was confined to CaMKII neurons in the ACA, with a lack of expression of mCherry in other areas throughout the brain. In a small portion of animals (5 out of 37 animals or 13.5%), limited uptake in dorsal portions of PL was noted in sparsely scattered cells (Figure 3A). CNO reduced neuronal activation in ACA, evidenced by significantly reduced mating-induced pERK expression in CNO-treated males compared with both vehicle-treated groups (Table S7). Surprisingly, CNO failed to reduce the numbers of Meth-induced cFos-expressing neurons and instead it was increased in CNO-treated males (Table S7). Finally, CNO treatments had no effect on numbers of CaMKII-expressing neurons (Table S7).

### Chemogenetic Inactivation of Ventral mPFC Did Not Affect Enhanced Drug-Seeking Behaviors After Concurrent Meth/Sex Experience

Since the validation study (Experiment 2) observed that ACA inactivation also reduced neuronal activation in PL and IL subregions of the mPFC (vmPFC), it was next tested whether this reduced vmPFC activity may have contributed to the effects of ACA CaMKII cell inactivation on attenuation of drug-seeking behavior during reinstatement. To accomplish this, vmPFC CaMKII neurons were chemogenetically inactivated during concurrent Meth/sex experience and subsequent drug seeking behavior was examined.

First, a small validation study (Experiment 4; experimental timeline shown in Figure 4A) was conducted to verify that CNO did not affect passive Meth-induced locomotor activity [*F*_(3,21)_ = 8.8, *p* < 0.001; Meth vs. saline within all CNO doses *p* = 0.0045–0.005; Figure 4F; see Table S8 for statistical analyses] nor initiation of mating behavior (Figure 4G). The localization and spread of DREADD expression is shown in Figures 4B–E. Next, in a separate cohort of animals and using a similar self-administration approach as for the ACA DREADD study (Experiment 5; experimental timeline shown in Figure 4H), it was demonstrated that inactivation of vmPFC CaMKII neurons with CNO did not affect active lever responding or Meth infusions earned (Figure 4I), total Meth intake (Table S10), or parameters of sexual behavior. In addition, inactivation of vmPFC neurons during the Meth/sex experience did not prevent concurrent Meth/sex-induced resistance to extinction [*F*_(1,126)_ = 5.2, *p* = 0.038; pairwise comparisons combined concurrent groups vs. non-concurrent: active responses Session 1 *p* = 0.001, Session 2 *p* = 0.011; Figure 4J]. Finally, inactivation of vmPFC during Meth/sex experience did not prevent or attenuate the effect of concurrent Meth/sex on increased relapse vulnerability in a cue-induced reinstatement test [*F*_(1,26)_ = 11.9, *p* = 0.002; Figure 4K]. Both vehicle- and CNO-pretreated concurrent groups, but not the vehicle non-concurrent group, significantly increased active responding during Cue Reinstatement (extinction vs. Cue Reinstatement within vehicle concurrent: *p* = 0.006, within CNO concurrent: *p* = 0.019; Figure 4K) compared to the average of active responses during the last 3 extinction sessions. However, in contrast to the ACA DREADD study the vehicle and CNO concurrent groups did not differ in reinstatement active responding during Cue Reinstatement (Figure 4K).

A week after the last reinstatement test, animals received CNO or vehicle and Meth-induced cFos (1 mg/kg, passive administration) and mating-induced pERK expression were analyzed. mCherry labeling was as in Figures 4B–E. CNO-treated males had significantly lower numbers of mating-induced pERK-expressing neurons in vmPFC compared with both vehicle-treated groups (Table S9). However, consistent with the finding in the ACA experiment above, numbers of Meth-induced cFos-expressing neurons were not significantly affected by CNO treatment (Table S9).

## Discussion

The current studies demonstrate that activity of ACA CaMKII neurons and their targets contributes to enhanced drug-seeking behavior in male rats following Meth exposures in a sexual context. Results fit with the overall framework that mPFC is involved in formation and consolidation of context-response associations, including a role for ACA in working memory for response-outcome associations (Kesner and Churchwell, 2011). Here, findings support a role for ACA in forming associations between an intrinsic context of Meth exposure while engaging in emotional reward behavior, which in this study consisted of sexual reward behavior (see Kuiper et al. (2019) for additional detailed discussion). Studies have also shown that inactivation of mPFC immediately after a learning task causes memory impairments (Carballo-Marquez et al., 2007). However, in the current study where the designer receptor was likely still activated after the task was completed (Vardy et al., 2015), there was no evidence of an overall memory deficit: acquisition of self-administration behavior and drug-seeking behavior during extinction were unaffected, suggesting that learning of Meth-cue associations remained intact. Importantly, ACA inactivation did not completely eliminate drug-seeking, but rather prevented the enhancement of drug-seeking during reinstatement observed after concurrent Meth and sex experience. Thus, findings here support a selective disruption of an associative learning between Meth and sexual behavior, without disruption of associative learning between Meth and associated cues. Notably, inactivation of ACA neurons during acquisition of concurrent Meth and sex experience prevented enhancement of drug-seeking during reinstatement, but not during extinction tests, which may instead be driven by other brain areas, such as ventral mPFC (Augur et al., 2016). Alternatively, ACA manipulations may have only affected expression of remote memories (Frankland et al., 2004; Teixeira et al., 2006; Restivo et al., 2009; Euston et al., 2012; Wartman and Holahan, 2014; Jin and Maren, 2015; Abate et al., 2018; Mashhoori et al., 2018) during reinstatement testing which occurred many days after CNO treatments, without affecting recent memory during the first extinction trials, which occurred within hours-to-days after last CNO treatment.

Several technical considerations were noted and validations provided for the chemogenetic approach utilized. First, a lack of off-target effects (MacLaren et al., 2016; Gomez et al., 2017; Manvich et al., 2018) was confirmed as there were no effects of DREADD-expressing virus or multiple dosages of CNO on locomotor or sexual behavior and no effects of DREADD-expressing virus alone on neural activity markers pERK and cFos. Many previous studies have demonstrated that the dosages of CNO used in the current studies have no effect on motivated behaviors, including drug self administration and reinstatement in animals with either control virus or missed injections (Augur et al., 2016; Padovan-Hernandez and Knackstedt, 2018; Mahler et al., 2019; O'Neal et al., 2019). A reduction of neural activation by CNO was confirmed for both Meth-induced cFos and mating-induced pERK in animals with no prior drug experience and using passive administration of Meth. However, confirmation of effects of CNO after completion of the operant self administration experiments, hence after repeated sex- and Meth-exposures (~3 weeks after last CNO and Meth injection), was less conclusive: CNO prevented mating-induced pERK, but Meth-induced cFos was not reduced. One possible explanation for the latter is that prior to these final tests, animals had been exposed to repeated Meth infusions and numerous behavioral tasks. Hence it is unclear if Meth-induced cFos was in the same neuronal ensemble of CaMKII cells as during the initial Meth exposures. Moreover, during this final test, vehicle-treated groups were not included, complicating a conclusive interpretation. Differences between neuron ensembles that are activated during Meth passive administration as in the first DREADD validation study vs. self administration in the subsequent studies are also a possibility. For example, additional neurons may be activated during self administration reflecting anticipation and arousal. However, such additional activation would have likely also been targeted by CNO in the current studies and thus did not appear to be a major caveat., Reduced activity of vmPFC neurons was observed as a result of inhibition of ACA CaMKII cells. This is unlikely a result of CNO action directly on ventral mPFC neurons, as mCherry-labeled cell bodies were not observed in these areas, but instead a result of connectivity between dorsal and ventral mPFC neuronal populations (Balfour et al., 2006; Hoover and Vertes, 2007). In agreement, a study using chemogenetic inhibition of ventral mPFC was conducted using methods similar to the ACA DREADD experiment, showing that inactivation of ventral mPFC CaMKII neurons during concurrent Meth and sex didn't prevent the enhancement of drug seeking behaviors during reinstatement tests or during extinction testing.

The main goal of the current studies was to test if activity of ACA CaMKII neurons contributes to the effects of concurrent Meth and sex on subsequent drug seeking behavior, but it was not tested if this contribution was specific only to the effects of Meth taking in a sexual context. In fact, we hypothesize and propose to examine in future studies that ACA CaMKII cell activity may play a role also in other factors that contribute to enhancement of drug seeking behavior, including extended Meth exposure. Hence, a separate experimental group receiving CNO while taking Meth without engaging in sexual behavior (non-concurrent) wasn't included in the current set of studies. The main reason for exclusion of this group was based on our previous findings that the limited Meth self administration failed to induce significant Meth seeking behavior during reinstatement testing (Kuiper et al., 2019). This was again observed in the vmPFC study. However, in the ACA DREADD study, a small, but significant increase in drug seeking was noted in the non-concurrent Meth group, in contrast to our previous studies. This slight enhancement is likely due to differences in the experimental design consisting of two additional Meth self-administration sessions prior to the five Meth and sex concurrent sessions that included CNO or vehicle treatments. Thus, current findings do not provide evidence that ACA CaMKII activity is exclusively critical to effects of Meth taking while engaging in sexual behavior on later drug seeking behavior. Furthermore, further experiments may determine whether concurrent social behavior or other natural rewards (including palatable food and wheel running), may be sufficient for the enhancement of drug-seeking behavior when presented following Meth administration and if so, whether ACA CaMKII cells mediate such effects.

An important future question is to determine the specific afferent projections by which the ACA regulates the effects of Meth and sex experience. ACA has dense projections to dorsal striatum (Gabbott et al., 2005), but also projects to NAc (albeit to a lesser extent; see Figure 2), and other mesocorticolimbic areas (Balfour et al., 2006). Current findings showed that inhibition of ACA CaMKII cells attenuated activity of NAc suggesting that ACA may regulate enhanced drug-seeking through the NAc, which regulates craving and relapse vulnerability (James et al., 2018), and exhibits plasticity in connection with increased drug-seeking (Scofield et al., 2016). Moreover, NAc neurons are co-activated by Meth and sex, as are basolateral amygdala neurons (Frohmader et al., 2010c). In addition, cue-driven drug-seeking is hypothesized to be modulated by dopamine signaling to the mPFC and BLA, which in turn influence the activity of NAc core (McFarland et al., 2003; Kalivas et al., 2005; Bossert et al., 2007, 2012; Lintas et al., 2012; Cruz et al., 2014; Keistler et al., 2017). Taken together, these findings highlight the important role of both the mPFC and BLA in fine-tuning the behavioral output elicited by cue-induced NAc dopamine efflux, such that dysfunction in these areas would lead to maladaptive behavior (Pelloux et al., 2013; Keistler et al., 2017; McGarry and Carter, 2017).

Another outstanding question is the exact mechanisms by which ACA CaMKII neurons drive the long-term effect of enhanced drug-seeking induced by concurrent Meth and sex, during which we hypothesize key molecular events occur to induce long-term plasticity associated with habitual, dysregulated drug-seeking (Nestler, 2008, 2014; Perrotti et al., 2008; Li et al., 2011; Muschamp et al., 2012; Godino et al., 2015; Zhang et al., 2018). Previously, we showed that concurrent Meth and sex experience induced long term increases in expression of phosphorylation of MAP kinase (pERK) in ACA CaMKII neurons (Kuiper et al., 2017), consistent with long term plasticity. The increased pERK expression was a synergistic effect of concurrent Meth and sex and not observed when Meth and sex were experienced non-concurrently (Kuiper et al., 2017). This plasticity may be a reflection of alterations in afferent signaling to the CaMKII cells, including local inhibitory interneurons (Morshedi and Meredith, 2007; Wearne et al., 2017). Cellular and epigenetic alterations have been observed in mPFC following chronic Meth (Howell and Kimmel, 2008; Shibasaki et al., 2011; Li et al., 2014) or other psychostimulants (Mychasiuk et al., 2013; Zhang et al., 2018). Since sexual behavior and drug exposure lead to similar molecular and plasticity mechanisms within NAc and VTA (Beloate and Coolen, 2017, 2018), it is interesting to test if concurrent Meth and sex may cause either additive or synergistic effects on such cellular alterations.

In conclusion, the current findings support the hypothesis that CaMKII neurons in the ACA play a critical role in establishing a conditioned association between Meth-taking and sexual behavior and subsequent enhancement of drug seeking behavior during reinstatement tests. Targeting the processes that mediate acquisition and subsequent stabilization of memory for concurrent Meth and sex experience is thus critical for understanding the robust drug-sex association and may potentially contribute to treatment targeted at unlearning these memories (Torregrossa and Taylor, 2013; Mohamed et al., 2017).

## Data Availability Statement

All datasets generated for this study are included in the article/Supplementary Material.

## Ethics Statement

The animal study was reviewed and approved by University of Mississippi Institutional Animal Care and Use Committee.

## Author Contributions

LK and LC designed the experiments. LK recorded and analyzed the data. KL and VM assisted in the surgical procedures and immunohistochemistry. LK and LC prepared the manuscript. All authors edited and finalized the manuscript.

## Conflict of Interest

The authors declare that the research was conducted in the absence of any commercial or financial relationships that could be construed as a potential conflict of interest.

## References

[B1] Abate G. Colazingari S. Accoto A. Conversi D. Bevilacqua A. (2018). Dendritic spine density and EphrinB2 levels of hippocampal and anterior cingulate cortex neurons increase sequentially during formation of recent and remote fear memory in the mouse. Behav. Brain Res. 344, 120–131. 10.1016/j.bbr.2018.02.011 29444449

[B2] Ahmadi K. Javadinia S. A. Saadat S. H. Ramezani M. A. Sedghijalal H. (2017). Triangular relationship among risky sexual behavior, addiction, and aggression: a systematic review. Electron. Physician 9, 5129–5137. 10.19082/5129 28979752 PMC5614302

[B3] Augur I. F. Wyckoff A. R. Aston-Jones G. Kalivas P. W. Peters J. (2016). Chemogenetic activation of an extinction neural circuit reduces cue-induced reinstatement of cocaine seeking. J. Neurosci. 36, 10174–10180. 10.1523/JNEUROSCI.0773-16.2016 27683912 PMC5039261

[B4] Balfour M. E. Brown J. L. Yu L. Coolen L. M. (2006). Potential contributions of efferents from medial prefrontal cortex to neural activation following sexual behavior in the male rat. J. Neurosci. 137, 1259–1276. 10.1016/j.neuroscience.2005.11.013 16388908

[B5] Beloate L. N. Coolen L. M. (2017). Influences of social reward experience on behavioral responses to drugs of abuse: review of shared and divergent neural plasticity mechanisms for sexual reward and drugs of abuse. Neurosci. Biobehav. Rev. 83, 356–372. 10.1016/j.neubiorev.2017.10.024 29108963

[B6] Beloate L. N. Coolen L. M. (2018). Effects of sexual experience on psychostimulant- and opiate-induced behavior and neural plasticity in the mesocorticolimbic pathway. Int. Rev. Neurobiol. 140, 249–270. 10.1016/bs.irn.2018.07.008 30193706

[B7] Beloate L. N. Omrani A. Adan R. A. Webb I. C. Coolen L. M. (2016). Ventral tegmental area dopamine cell activation during male rat sexual behavior regulates neuroplasticity and d-amphetamine cross-sensitization following sex abstinence. J. Neurosci. 36, 9949–9961. 10.1523/JNEUROSCI.0937-16.2016 27656032 PMC6705564

[B8] Berry M. S. Johnson M. W. (2018). Does being drunk or high cause HIV sexual risk behavior? A systematic review of drug administration studies. Pharmacol. Biochem. Behav. 164, 125–138. 10.1016/j.pbb.2017.08.009 28843425 PMC5747990

[B9] Bossert J. M. Marchant N. J. Calu D. J. Shaham Y. (2013). The reinstatement model of drug relapse: recent neurobiological findings, emerging research topics, and translational research. Psychopharmacology (Berl). 229, 453–476. 10.1007/s00213-013-3120-y 23685858 PMC3770775

[B10] Bossert J. M. Poles G. C. Wihbey K. A. Koya E. Shaham Y. (2007). Differential effects of blockade of dopamine D1-family receptors in nucleus accumbens core or shell on reinstatement of heroin seeking induced by contextual and discrete cues. J. Neurosci. 27, 12655–12663. 10.1523/JNEUROSCI.3926-07.2007 18003845 PMC2117350

[B11] Bossert J. M. Stern A. L. Theberge F. R. Marchant N. J. Wang H. L. Morales M.. (2012). Role of projections from ventral medial prefrontal cortex to nucleus accumbens shell in context-induced reinstatement of heroin seeking. J. Neurosci. 32, 4982–4991. 10.1523/JNEUROSCI.0005-12.2012 22492053 PMC3335169

[B12] Campbell E. J. Marchant N. J. (2018). The use of chemogenetics in behavioural neuroscience: receptor variants, targeting approaches and caveats. Br. J. Pharmacol. 175, 994–1003. 10.1111/bph.14146 29338070 PMC5843707

[B13] Carballo-Marquez A. Vale-Martinez A. Guillazo-Blanch G. Torras-Garcia M. Boix-Trelis N. Marti-Nicolovius M. (2007). Differential effects of muscarinic receptor blockade in prelimbic cortex on acquisition and memory formation of an odor-reward task. Learn. Mem. 14, 616–624. 10.1101/lm.597507 17848501 PMC1994078

[B14] Cho A. K. Melega W. P. Kuczenski R. Segal D. S. (2001). Relevance of pharmacokinetic parameters in animal models of methamphetamine abuse. Synapse 39, 161–166. 10.1002/1098-2396(200102)39:2<161::AID-SYN7>3.0.CO;2-E 11180503

[B15] Cruz F. C. Babin K. R. Leao R. M. Goldart E. M. Bossert J. M. Shaham Y.. (2014). Role of nucleus accumbens shell neuronal ensembles in context-induced reinstatement of cocaine-seeking. J. Neurosci. 34, 7437–7446. 10.1523/JNEUROSCI.0238-14.2014 24872549 PMC4035511

[B16] Davis J. F. Loos M. di Sebastiano A. R. Brown J. L. Lehman M. N. Coolen L. M. (2010). Lesions of the medial prefrontal cortex cause maladaptive sexual behavior in male rats. Biol. Psychiatr. 67, 1199–1204. 10.1016/j.biopsych.2009.12.029 20346444 PMC2908911

[B17] de Wit H. Sayette M. (2018). Considering the context: social factors in responses to drugs in humans. Psychopharmacology (Berl). 235, 935–945. 10.1007/s00213-018-4854-3 29470605 PMC5871591

[B18] Euston D. R. Gruber A. J. McNaughton B. L. (2012). The role of medial prefrontal cortex in memory and decision making. Neuron 76, 1057–1070. 10.1016/j.neuron.2012.12.002 23259943 PMC3562704

[B19] Fanous S. Guez-Barber D. H. Goldart E. M. Schrama R. Theberge F. R. Shaham Y.. (2013). Unique gene alterations are induced in FACS-purified Fos-positive neurons activated during cue-induced relapse to heroin seeking. J. Neurochem. 124, 100–108. 10.1111/jnc.12074 23113797 PMC3518644

[B20] Frankland P. W. Bontempi B. Talton L. E. Kaczmarek L. Silva A. J. (2004). The involvement of the anterior cingulate cortex in remote contextual fear memory. Science 304, 881–883. 10.1126/science.1094804 15131309

[B21] Frohmader K. S. Bateman K. L. Lehman M. N. Coolen L. M. (2010a). Effects of methamphetamine on sexual performance and compulsive sex behavior in male rats. Psychopharmacology (Berl). 212, 93–104. 10.1007/s00213-010-1930-8 20623108

[B22] Frohmader K. S. Lehman M. N. Laviolette S. R. Coolen L. M. (2011). Concurrent exposure to methamphetamine and sexual behavior enhances subsequent drug reward and causes compulsive sexual behavior in male rats. J. Neurosci. 31, 16473–16482. 10.1523/JNEUROSCI.4013-11.2011 22072697 PMC6633219

[B23] Frohmader K. S. Pitchers K. K. Balfour M. E. Coolen L. M. (2010b). Mixing pleasures: review of the effects of drugs on sex behavior in humans and animal models. Horm. Behav. 58, 149–162. 10.1016/j.yhbeh.2009.11.009 20004662

[B24] Frohmader K. S. Wiskerke J. Wise R. A. Lehman M. N. Coolen L. M. (2010c). Methamphetamine acts on subpopulations of neurons regulating sexual behavior in male rats. J. Neurosci. 166, 771–784. 10.1016/j.neuroscience.2009.12.070 20045448 PMC2837118

[B25] Gabbott P. L. Warner T. A. Jays P. R. Salway P. Busby S. J. (2005). Prefrontal cortex in the rat: projections to subcortical autonomic, motor, and limbic centers. J. Comp. Neurol. 492, 145–177. 10.1002/cne.20738 16196030

[B26] Gholizadeh S. Sun N. De Jaeger X. Bechard M. Coolen L. Laviolette S. R. (2013). Early versus late-phase consolidation of opiate reward memories requires distinct molecular and temporal mechanisms in the amygdala-prefrontal cortical pathway. PLoS ONE 8:e63612. 10.1371/journal.pone.0063612 23696837 PMC3656057

[B27] Godino A. Jayanthi S. Cadet J. L. (2015). Epigenetic landscape of amphetamine and methamphetamine addiction in rodents. Epigenetics 10, 574–580. 10.1080/15592294.2015.1055441 26023847 PMC4622560

[B28] Gomez J. L. Bonaventura J. Lesniak W. Mathews W. B. Sysa-Shah P. Rodriguez L. A.. (2017). Chemogenetics revealed: DREADD occupancy and activation via converted clozapine. Science 357, 503–507. 10.1126/science.aan2475 28774929 PMC7309169

[B29] Heilig M. Epstein D. H. Nader M. A. Shaham Y. (2016). Time to connect: bringing social context into addiction neuroscience. Nat. Rev. Neurosci. 17, 592–599. 10.1038/nrn.2016.67 27277868 PMC5523661

[B30] Hoover W. B. Vertes R. P. (2007). Anatomical analysis of afferent projections to the medial prefrontal cortex in the rat. Brain Struct. Funct. 212, 149–179. 10.1007/s00429-007-0150-4 17717690

[B31] Howell L. L. Kimmel H. L. (2008). Monoamine transporters and psychostimulant addiction. Biochem. Pharmacol. 75, 196–217. 10.1016/j.bcp.2007.08.003 17825265

[B32] James M. H. McGlinchey E. M. Vattikonda A. Mahler S. V. Aston-Jones G. (2018). Cued reinstatement of cocaine but not sucrose seeking is dependent on dopamine signaling in prelimbic cortex and is associated with recruitment of prelimbic neurons that project to contralateral nucleus accumbens core. Int. J. Neuropsychopharmacol. 21, 89–94. 10.1093/ijnp/pyx107 29165565 PMC5789262

[B33] Jin J. Maren S. (2015). Prefrontal-hippocampal interactions in memory and emotion. Front. Syst. Neurosci. 9:170. 10.3389/fnsys.2015.00170 26696844 PMC4678200

[B34] Kalivas P. W. Volkow N. Seamans J. (2005). Unmanageable motivation in addiction: a pathology in prefrontal-accumbens glutamate transmission. Neuron 45, 647–650. 10.1016/j.neuron.2005.02.005 15748840

[B35] Katzman A. Alberini C. M. (2018). NLGN1 and NLGN2 in the prefrontal cortex: their role in memory consolidation and strengthening. Curr. Opin. Neurobiol. 48, 122–130. 10.1016/j.conb.2017.12.003 29278843 PMC5825275

[B36] Keistler C. R. Hammarlund E. Barker J. M. Bond C. W. DiLeone R. J. Pittenger C.. (2017). Regulation of alcohol extinction and cue-induced reinstatement by specific projections among medial prefrontal cortex, nucleus accumbens, and basolateral amygdala. J. Neurosci. 37, 4462–4471. 10.1523/JNEUROSCI.3383-16.2017 28336571 PMC5413184

[B37] Kesner R. P. Churchwell J. C. (2011). An analysis of rat prefrontal cortex in mediating executive function. Neurobiol. Learn. Mem. 96, 417–431. 10.1016/j.nlm.2011.07.002 21855643

[B38] Kuiper L. B. Beloate L. N. Dupuy B. M. Coolen L. M. (2019). Drug-taking in a socio-sexual context enhances vulnerability for addiction in male rats. Neuropsychopharmacology 44, 503–513. 10.1038/s41386-018-0235-1 30337639 PMC6333843

[B39] Kuiper L. B. Coolen L. M. (2018). Compulsive sexual behavior in humans and preclinical models. Curr. Sex Health Rep 44, 503–513. 10.1007/s11930-018-0157-230337639

[B40] Kuiper L. B. Frohmader K. S. Coolen L. M. (2017). Maladaptive sexual behavior following concurrent methamphetamine and sexual experience in male rats is associated with altered neural activity in frontal cortex. Neuropsychopharmacology 42, 2011–2020. 10.1038/npp.2017.1 28051103 PMC5561340

[B41] Lacy R. T. Strickland J. C. Feinstein M. A. Robinson A. M. Smith M. A. (2016). The effects of sex, estrous cycle, and social contact on cocaine and heroin self-administration in rats. Psychopharmacology (Berl). 233, 3201–3210. 10.1007/s00213-016-4368-9 27370020 PMC5259804

[B42] Lennette D. A. (1978). An improved mounting medium for immunofluorescence microscopy. Am. J. Clin. Pathol. 69, 647–648. 27089 10.1093/ajcp/69.6.647

[B43] Li H. Li F. Wu N. Su R. B. Li J. (2014). Methamphetamine induces dynamic changes of histone deacetylases in different phases of behavioral sensitization. CNS Neurosci. Ther. 20, 874–876. 10.1111/cns.12301 24954603 PMC6493015

[B44] Li Y. Q. Xue Y. X. He Y. Y. Li F. Q. Xue L. F. Xu C. M.. (2011). Inhibition of PKMzeta in nucleus accumbens core abolishes long-term drug reward memory. J. Neurosci. 31, 5436–5446. 10.1523/JNEUROSCI.5884-10.2011 21471379 PMC3150199

[B45] Lintas A. Chi N. Lauzon N. M. Bishop S. F. Sun N. Tan H. (2012). Inputs from the basolateral amygdala to the nucleus accumbens shell control opiate reward magnitude via differential dopamine D1 or D2 receptor transmission. Eur. J. Neurosci. 35, 279–290. 10.1111/j.1460-9568.2011.07943.x 22236063

[B46] Lorvick J. Bourgois P. Wenger L. D. Arreola S. G. Lutnick A. Wechsberg W. M.. (2012). Sexual pleasure and sexual risk among women who use methamphetamine: a mixed methods study. Int. J. Drug Policy 23, 385–392. 10.1016/j.drugpo.2012.07.005 22954501 PMC3466046

[B47] MacLaren D. A. Browne R. W. Shaw J. K. Krishnan Radhakrishnan S. Khare P. Espana R.. (2016). Clozapine N-oxide administration produces behavioral effects in long-evans rats: implications for designing DREADD experiments. eNeuro 3. 10.1523/ENEURO.0219-16.2016 27822508 PMC5089539

[B48] Mahler S. V. Brodnik Z. D. Cox B. M. Buchta W. C. Bentzley B. S. Quintanilla J.. (2019). Chemogenetic manipulations of ventral tegmental area dopamine neurons reveal multifaceted roles in cocaine abuse. J. Neurosci. 39, 503–518. 10.1523/JNEUROSCI.0537-18.2018 30446532 PMC6335749

[B49] Manvich D. F. Webster K. A. Foster S. L. Farrell M. S. Ritchie J. C. Porter J. H.. (2018). The DREADD agonist clozapine N-oxide (CNO) is reverse-metabolized to clozapine and produces clozapine-like interoceptive stimulus effects in rats and mice. Sci. Rep. 8:3840. 10.1038/s41598-018-22116-z 29497149 PMC5832819

[B50] Mashhoori A. Hashemnia S. McNaughton B. L. Euston D. R. Gruber A. J. (2018). Rat anterior cingulate cortex recalls features of remote reward locations after disfavoured reinforcements. Elife 7. 10.7554/eLife.29793 29664400 PMC5931797

[B51] McFarland K. Lapish C. C. Kalivas P. W. (2003). Prefrontal glutamate release into the core of the nucleus accumbens mediates cocaine-induced reinstatement of drug-seeking behavior. J. Neurosci. 23, 3531–3537. 10.1523/JNEUROSCI.23-08-03531.2003 12716962 PMC6742291

[B52] McGarry L. M. Carter A. G. (2017). Prefrontal cortex drives distinct projection neurons in the basolateral Amygdala. Cell Rep. 21, 1426–1433. 10.1016/j.celrep.2017.10.046 29117549 PMC5714295

[B53] Mohamed R. M. P. Kumar J. Yap E. Mohamed I. N. Sidi H. Adam R. L.. (2017). Try to remember: interplay between memory and substance use disorder. Curr. Drug Targets 10.2174/1389450118666170622092824 28641520

[B54] Morici J. F. Bekinschtein P. Weisstaub N. V. (2015). Medial prefrontal cortex role in recognition memory in rodents. Behav. Brain Res. 292, 241–251. 10.1016/j.bbr.2015.06.030 26115848

[B55] Morshedi M. M. Meredith G. E. (2007). Differential laminar effects of amphetamine on prefrontal parvalbumin interneurons. Neuroscience 149, 617–624. 10.1016/j.neuroscience.2007.07.047 17931790 PMC2447530

[B56] Muschamp J. W. Nemeth C. L. Robison A. J. Nestler E. J. Carlezon W. A. Jr. (2012). DeltaFosB enhances the rewarding effects of cocaine while reducing the pro-depressive effects of the kappa-opioid receptor agonist U50488. Biol. Psychiatry 71, 44–50. 10.1016/j.biopsych.2011.08.011 21962331 PMC3230776

[B57] Mychasiuk R. Muhammad A. Ilnytskyy S. Kolb B. (2013). Persistent gene expression changes in NAc, mPFC, and OFC associated with previous nicotine or amphetamine exposure. Behav. Brain Res. 256, 655–661. 10.1016/j.bbr.2013.09.006 24021241

[B58] Nestler E. J. (2008). Review. transcriptional mechanisms of addiction: role of DeltaFosB. Philos. Trans. R. Soc. Lond. B Biol. Sci. 363, 3245–3255. 10.1098/rstb.2008.0067 18640924 PMC2607320

[B59] Nestler E. J. (2014). Epigenetic mechanisms of drug addiction. Neuropharmacology 76(Pt B), 259–268. 10.1016/j.neuropharm.2013.04.004 23643695 PMC3766384

[B60] O'Neal T. J. Nooney M. N. Thien K. Ferguson S. M. (2019). Chemogenetic modulation of accumbens direct or indirect pathways bidirectionally alters reinstatement of heroin-seeking in high- but not low-risk rats. Neuropsychopharmacology. 10.1038/s41386-019-0571-9 PMC7297977 31747681

[B61] Padovan-Hernandez Y. Knackstedt L. A. (2018). Dose-dependent reduction in cocaine-induced locomotion by clozapine-N-oxide in rats with a history of cocaine self-administration. Neurosci. Lett. 674, 132–135. 10.1016/j.neulet.2018.03.045 29571824 PMC5899660

[B62] Pelloux Y. Murray J. E. Everitt B. J. (2013). Differential roles of the prefrontal cortical subregions and basolateral amygdala in compulsive cocaine seeking and relapse after voluntary abstinence in rats. Eur. J. Neurosci. 38, 3018–3026. 10.1111/ejn.12289 23815783 PMC3910160

[B63] Perrotti L. I. Weaver R. R. Robison B. Renthal W. Maze I. Yazdani S.. (2008). Distinct patterns of DeltaFosB induction in brain by drugs of abuse. Synapse 62, 358–369. 10.1002/syn.20500 18293355 PMC2667282

[B64] Peters G. J. David C. N. Marcus M. D. Smith D. M. (2013). The medial prefrontal cortex is critical for memory retrieval and resolving interference. Learn. Mem. 20, 201–209. 10.1101/lm.029249.112 23512936 PMC3604648

[B65] Restivo L. Vetere G. Bontempi B. Ammassari-Teule M. (2009). The formation of recent and remote memory is associated with time-dependent formation of dendritic spines in the hippocampus and anterior cingulate cortex. J. Neurosci. 29, 8206–8214. 10.1523/JNEUROSCI.0966-09.2009 19553460 PMC6666032

[B66] Riga D. Matos M. R. Glas A. Smit A. B. Spijker S. Van den Oever M. C. (2014). Optogenetic dissection of medial prefrontal cortex circuitry. Front. Syst. Neurosci. 8:230. 10.3389/fnsys.2014.00230 25538574 PMC4260491

[B67] Riviere G. J. Gentry W. B. Owens S. M. (2000). Disposition of methamphetamine and its metabolite amphetamine in brain and other tissues in rats after intravenous administration. J. Pharmacol. Exp. Ther. 292, 1042–1047. 10688621

[B68] Robinson A. M. Fronk G. E. Zhang H. Tonidandel S. Smith M. A. (2017). The effects of social contact on cocaine intake in female rats. Drug Alcohol Depend 177, 48–53. 10.1016/j.drugalcdep.2017.03.027 28558271 PMC5534368

[B69] Robinson A. M. Lacy R. T. Strickland J. C. Magee C. P. Smith M. A. (2016). The effects of social contact on cocaine intake under extended-access conditions in male rats. Exp. Clin. Psychopharmacol. 24, 285–296. 10.1037/pha0000078 27454676 PMC4965182

[B70] Safi M. H. Younesi S. J. Dadkhah A. Farhoudian A. Fallahi-Khoshknab M. Azkhosh M. (2016). The role of sexual behaviors in the relapse process in iranian methamphetamine users: a qualitative study. Addict Health 8, 242–251. 28819555 PMC5554804

[B71] Saw Y. M. Saw T. N. Chan N. Cho S. M. Jimba M. (2018). Gender-specific differences in high-risk sexual behaviors among methamphetamine users in Myanmar-China border city, Muse, Myanmar: who is at risk? BMC Public Health 18:209. 10.1186/s12889-018-5113-6 29390989 PMC5796492

[B72] Schmidt C. Morris L. S. Kvamme T. L. Hall P. Birchard T. Voon V. (2017). Compulsive sexual behavior: prefrontal and limbic volume and interactions. Hum. Brain Mapp. 38, 1182–1190. 10.1002/hbm.23447 27787929 PMC5324617

[B73] Scofield M. D. Heinsbroek J. A. Gipson C. D. Kupchik Y. M. Spencer S. Smith A. C.. (2016). The nucleus accumbens: mechanisms of addiction across drug classes reflect the importance of glutamate homeostasis. Pharmacol. Rev. 68, 816–871. 10.1124/pr.116.012484 27363441 PMC4931870

[B74] Shibasaki M. Mizuno K. Kurokawa K. Ohkuma S. (2011). L-type voltage-dependent calcium channels facilitate acetylation of histone H3 through PKCgamma phosphorylation in mice with methamphetamine-induced place preference. J. Neurochem. 118, 1056–1066. 10.1111/j.1471-4159.2011.07387.x 21781114

[B75] Stern C. A. Gazarini L. Vanvossen A. C. Hames M. S. Bertoglio L. J. (2014). Activity in prelimbic cortex subserves fear memory reconsolidation over time. Learn. Mem. 21, 14–20. 10.1101/lm.032631.113 24344180 PMC3867715

[B76] Teixeira C. M. Pomedli S. R. Maei H. R. Kee N. Frankland P. W. (2006). Involvement of the anterior cingulate cortex in the expression of remote spatial memory. J. Neurosci. 26, 7555–7564. 10.1523/JNEUROSCI.1068-06.2006 16855083 PMC6674278

[B77] Torregrossa M. M. Taylor J. R. (2013). Learning to forget: manipulating extinction and reconsolidation processes to treat addiction. Psychopharmacology (Berl). 226, 659–672. 10.1007/s00213-012-2750-9 22638814 PMC3466391

[B78] Tull M. T. Gratz K. L. Weiss N. H. (2011). Exploring associations between borderline personality disorder, crack/cocaine dependence, gender, and risky sexual behavior among substance-dependent inpatients. Personal. Disord. 2, 209–219. 10.1037/a0021878 22448767

[B79] Urban D. J. Roth B. L. (2015). DREADDs (designer receptors exclusively activated by designer drugs): chemogenetic tools with therapeutic utility. Annu. Rev. Pharmacol. Toxicol. 55, 399–417. 10.1146/annurev-pharmtox-010814-124803 25292433

[B80] Vardy E. Robinson J. E. Li C. Olsen R. H. J. DiBerto J. F. Giguere P. M.. (2015). A new DREADD facilitates the multiplexed chemogenetic interrogation of behavior. Neuron 86, 936–946. 10.1016/j.neuron.2015.03.065 25937170 PMC4441592

[B81] Venniro M. Russell T. I. Zhang M. Shaham Y. (2019). Operant social reward decreases incubation of heroin craving in male and female rats. Biol. Psychiatry 86, 848–856. 10.1016/j.biopsych.2019.05.018 31326085 PMC8383184

[B82] Venniro M. Zhang M. Caprioli D. Hoots J. K. Golden S. A. Heins C.. (2018). Volitional social interaction prevents drug addiction in rat models. Nat. Neurosci. 21, 1520–1529. 10.1038/s41593-018-0246-6 30323276 PMC7386559

[B83] Voon V. Mole T. B. Banca P. Porter L. Morris L. Mitchell S.. (2014). Neural correlates of sexual cue reactivity in individuals with and without compulsive sexual behaviours. PLoS ONE 9:e102419. 10.1371/journal.pone.0102419 25013940 PMC4094516

[B84] Wartman B. C. Holahan M. R. (2014). The impact of multiple memory formation on dendritic complexity in the hippocampus and anterior cingulate cortex assessed at recent and remote time points. Front. Behav. Neurosci. 8:128. 10.3389/fnbeh.2014.00128 24795581 PMC4001003

[B85] Wearne T. A. Parker L. M. Franklin J. L. Goodchild A. K. Cornish J. L. (2017). Behavioral sensitization to methamphetamine induces specific interneuronal mRNA pathology across the prelimbic and orbitofrontal cortices. Prog. Neuropsychopharmacol. Biol. Psychiatry 77, 42–48. 10.1016/j.pnpbp.2017.03.018 28351548

[B86] Zhang Y. X. Akumuo R. C. Espana R. A. Yan C. X. Gao W. J. Li Y. C. (2018). The histone demethylase KDM6B in the medial prefrontal cortex epigenetically regulates cocaine reward memory. Neuropharmacology 141, 113–125. 10.1016/j.neuropharm.2018.08.030 30165076 PMC6170674

