# Supplementary material for: Enhancement of Drug Seeking Following Drug Taking in a Sexual Context Requires Anterior Cingulate Cortex Activity in Male Rats
Source: Front Behav Neurosci. 2020 Jun 25;14:87. doi: 10.3389/fnbeh.2020.00087 (PMC7330085; doi:10.3389/fnbeh.2020.00087)
Supplement: Supplementary file 1 [file Data_Sheet_1.docx]

***Supplementary Material***

| **Figure #** | **Measure** | **Statistical Test** | **Factor** | **F value/t value** | **Main effect** | **Pairwise Comparison(s)** | **Pairwise effects** |
| --- | --- | --- | --- | --- | --- | --- | --- |
| 1F | cFos per mm^2^ | 2-way ANOVA | Meth | F(1,15) = 12.1 | P = 0.003 | Within sex-treated, Meth vs saline | P = 0.007 |
|  |  |  |  |  |  | Within no sex-treated, Meth vs saline | P = 0.025 |
| 1G | pERK per mm^2^ | 2-way ANOVA | sex | F(1,15) = 20.5 | P < 0.001 | Within Meth-treated, sex vs no sex | P = 0.021 |
|  |  |  |  |  |  | Within saline-treated, sex vs no sex | P < 0.001 |
| 1H | c-Fos + pERK (double-labeled) per mm^2^ | 2-way ANOVA | Meth | F(1,15) = 11.9 | P = 0.004 | Within sex-treated, Meth vs saline | P < 0.001 |
|  |  |  | sex | F(1,15) = 16.6 | P = 0.001 | Within Meth-treated, sex vs no sex | P < 0.001 |
|  |  |  | Meth x sex | F(1,15) = 4.9 | P = 0.043 | -- | -- |

**Table S1.** Detailed statistical analysis information for all results reported in main text for Figure 1. Meth/Sex (n=4), Meth/No Sex (n=5), Saline/Sex (n=5), and Saline/No Sex (n=5).

| **Figure #** | **Measure** | **Statistical Test** | **Factor** | **F value/t value** | **Main effect** | **Pairwise Comparison(s)** | **Pairwise effects** |
| --- | --- | --- | --- | --- | --- | --- | --- |
| 2F | Distance traveled (cm) | 2-way RM ANOVA | group | F(7,40) = 17.7 | P < 0.001 | all CNO doses, Meth vs saline | P = 0.002 - < 0.001 |
| 2H | ACA cFos | 2-way ANOVA | CNO | F(1,10) = 8.6 | P = 0.015 | within Meth | P < 0.001 |
|  |  |  | Meth | F(1,10) = 14.5 | P = 0.003 | within veh | P < 0.001 |
|  |  |  | CNO x Meth | F(1,10) = 16.5 | P = 0.002 | -- | -- |
|  | PL cFos | 2-way ANOVA | CNO | F(1,10) = 5.9 | P = 0.036 | within Meth | P = 0.002 |
|  |  |  | Meth | F(1,10) = 12.0 | P = 0.006 | within veh | P < 0.001 |
|  |  |  | CNO x Meth | F(1,10) = 8.3 | P = 0.016 | -- | -- |
|  | IL cFos | 2-way ANOVA | CNO | F(1,10) = 51.9 | P < 0.001 | within Meth | P < 0.001 |
|  |  |  |  |  |  | within sal | P = 0.042 |
|  |  |  | Meth | F(1,10) = 54.1 | P < 0.001 | within veh | P < 0.001 |
|  |  |  |  |  |  | within CNO | P = 0.035 |
|  |  |  | CNO x Meth | F(1,10) = 20.1 | P = 0.001 | -- | -- |
| 2I | NAc core cFos | 2-way ANOVA | CNO | F(1,10) = 6.4 | P = 0.030 | within Meth | P = 0.023 |
|  |  |  | Meth | F(1,10) = 8.2 | P = 0.017 | within veh | P = 0.009 |
|  | NAc shell cFos | 2-way ANOVA | CNO | F(1,10) = 11.2 | P = 0.007 | within Meth | P = 0.002 |
|  |  |  | Meth | F(1,10) = 14.7 | P = 0.003 | within veh | P = 0.002 |
| 2J | ACA pERK | 2-way ANOVA | CNO | F(1,9) = 9.1 | P = 0.015 | within sex-treated | P < 0.001 |
|  |  |  | sex | F(1,9) = 25.1 | P < 0.001 | within veh | P < 0.001 |
|  |  |  | CNO x sex | F(1,9) = 11.1 | P = 0.009 | -- | -- |
|  | PL pERK | 2-way ANOVA | CNO | F(1,9) = 33.2 | P < 0.001 | within sex-treated | P < 0.001 |
|  |  |  | sex | F(1,9) = 6.1 | P = 0.36 | within veh | P = 0.002 |
|  |  |  | CNO x sex | F(1,9) = 7.6 | P = 0.022 | -- | -- |
|  | IL pERK | 2-way ANOVA | CNO | F(1,9) = 60.6 | P < 0.001 | Within sex-treated | P < 0.001 |
|  |  |  | sex | F(1,9) = 23.8 | P < 0.001 | within veh | P < 0.001 |
|  |  |  | CNO x sex | F(1,9) = 6.0 | P = 0.037 | -- |  |
| 2K | NAc core pERK | 2-way ANOVA | CNO | F(1,9) = 31.9 | P < 0.001 | Within sex-treated | P < 0.001 |
|  |  |  | sex | F(1,9) = 67.0 | P < 0.001 | within veh | P < 0.001 |
|  |  |  | CNO x sex | F(1,9) = 39.0 | P < 0.001 | -- | -- |
|  | NAc shell pERK | 2-way ANOVA | CNO | F(1,9) = 16.7 | P = 0.003 | Within sex-treated | P = 0.001 |
|  |  |  | sex | F(1,9) = 11.1 | P = 0.009 | within veh | P = 0.001 |
|  |  |  | CNO x sex | F(1,9) = 6.0 | P 0.036 | -- | -- |

**Table S2.** Detailed statistical analysis information for all results reported in main text for Figure 2. CNO (1 mg/kg)/Meth/Sex (n=4), CNO (1 mg/kg)/Sal/No Sex (n=4), Veh/Meth/Sex (n=4), and Veh/Sal/No Sex (n=4), CNO (0.5 mg/kg)/Meth/Sex (n=3), CNO (0.5 mg/kg)/Sal/No Sex (n=3), CNO (3 mg/kg)/Meth/Sex (n=3), CNO (3 mg/kg)/Sal/No Sex (n=3).

| **Group** | **POA**  **# pERK cells/mm^2^** | **VP**  **# cFos cells/mm^2^** |
| --- | --- | --- |
| veh + sal + no sex | 24.3 ± 7.9 | 32.8 ± 1.6 |
| veh + Meth + sex | 68.8 ± 7.7* | 135.9 ± 7.0* |
| CNO + sal + no sex | 13.0 ± 3.5 | 18.8 ± 6.4 |
| CNO + Meth + sex | 63.7 ± 6.4* | 137.5 ± 15.3* |

**Table S3.** Mean ± SEM numbers of pERK-immunoreactive cells per square mm in the medial preoptic area (POA) and cFos-immunoreactive cells in the ventral pallidum (VP) in the 4 groups (n=4 per group) from the passive administration ACA DREADD experiment. *indicates significant difference vs no sex control group within veh or CNO-treated groups; Mating significantly induced pERK in POA (within veh p=0.007, within CNO p=0.001) and sex significantly induced cFos in VP (within veh p<0.001, within CNO p<0.001), while CNO had no effects compared to vehicle-treated groups. POA: preoptic area; VP: ventral pallidum.

|  |  |  | |
| --- | --- | --- | --- |
|  | **TOTAL INFUSIONS EARNED** | **TOTAL METH INTAKE (mg/kg)** | |
| Veh concurrent | 149.8 ± 9.6 | | 6.0 ± 0.385 |
| Veh non-concurrent | 139.0 ± 20.2 | | 5.6 ± 0.809 |
| CNO concurrent | 152.5 ± 7.0 | | 6.1 ± 0.292 |

**Table S4.** Average Meth intake across drug self-administration and chemogenetic inactivation of ACA; veh: vehicle, CNO: clozapine-*N*-oxide, Meth: methamphetamine. No significant differences were detected between groups. All data are expressed as Mean ± SEM.

|  | Session |  |  |  |  |  |
| --- | --- | --- | --- | --- | --- | --- |
|  | SA1 | SA2 | SA3 | SA4 | SA5 | Perfusion Day |
| **Vehicle NC** |  |  |  |  |  |  |
| ML | 305.8 ± 103.4 | 372.5 ± 177.4 | 407.2 ± 246.6 | 304.0 ± 158.0 | 239.0 ± 209.4 | 66.3 ± 28.2 |
| IL | 938.5 ± 679.4 | 321.0 ± 198.6 | 570.8 ± 297.1 | 162.0 ± 88.8 | 83.8 ± 45.9 | 69.7 ± 27.3 |
| EL | 1166.7 ± 238.5 | 688.8 ± 112.7 | 612.0 ± 16.8 | 735.8 ± 175.9 | 621.5 ± 142.2 | N/A |
| # mounts | 15.0 ± 4.5 | 13.7 ± 3.7 | 15.0 ± 2.6 | 6.4 ± 1.4 | 9.2 ± 2.1 | 4.0 ± 0.4 |
| # intromissions | 14.5 ± 5.6 | 15.7 ± 3.5 | 16.8 ± 4.3 | 20.5 ± 3.1 | 24.5 ± 5.7 | 6.5 ± 1.1 |
| Cop. eff. | 42.6 ± 10.1 | 52.0 ± 11.9 | 49.4 ± 10.3 | 60.0 ± 15.1 | 60.4 ± 15.5 | 59.7 ± 4.5 |
| **Vehicle C** |  |  |  |  |  |  |
| ML | 358.1 ± 150.3 | 145.3 ± 56.3 | 271.5 ± 184.8 | 194.6 ± 81.5 | 78.0 ± 20.4 | 103.9 ± 42.9 |
| IL | 361.2 ± 149.8 | 148.9 ± 56.7 | 286.5 ± 183.1 | 253.6 ± 91.5 | 120.5 ± 29.0 | 114.5 ± 46.4 |
| EL | 607.1 ± 60.4 | 552.4 ± 59.0 | 454.7 ± 47.5 | 455.5 ± 70.5 | 442.5 ± 65.9 | N/A |
| # mounts | 6.5 ± 1.4 | 14.0 ± 3.7 | 13.3 ± 3.5 | 10.8 ± 2.8 | 14.1 ± 4.7 | 7.6 ± 1.4 |
| # intromissions | 15.5 ± 1.6 | 16.7 ± 1.0 | 14.5 ± 1.0 | 14.3 ± 2.3 | 15.0 ± 1.9 | 6.8 ± 1.3 |
| Cop. eff. | 73.3 ± 3.7 | 59.8 ± 5.3 | 58.3 ± 4.6 | 55.9 ± 6.0 | 57.4 ± 4.5 | 47.2 ± 6.9 |
| **CNO C** |  |  |  |  |  |  |
| ML | 482.9 ± 211.1 | 326.2 ± 131.9 | 159.3 ± 47.9 | 282.3 ± 143.5 | 143.2 ± 54.9 | 80.8 ± 39.1 |
| IL | 511.3 ± 206.3 | 370.3 ± 133.9 | 170.4 ± 47.4 | 292.5 ± 142.7 | 145.6 ± 54.7 | 108.9 ± 37.9 |
| EL | 1221.9 ± 298.9 | 729.3 ± 99.1 | 582.4 ± 85.5 | 503.5 ± 67.7 | 521.8 ± 88.0 | N/A |
| # mounts | 14.9 ± 3.9 | 14.5 ± 4.5 | 23.3 ± 9.5 | 11.8 ± 2.5 | 11.7 ± 2.1 | 7.3 ± 1.7 |
| # intromissions | 14.1 ± 2.6 | 15.5 ± 2.0 | 16.3 ± 1.6 | 14.4 ± 1.7 | 15.3 ± 2.5 | 7.1 ± 1.3 |
| Cop. eff. | 53.2 ± 6.4 | 58.9 ± 5.5 | 55.8 ± 5.7 | 59.4 ± 5.1 | 58.4 ± 5.6 | 50.6 ± 6.2 |

**Table S5.** Mean ± SEM behavioral measures for the three groups (veh NC n=6, veh C n=12, CNO C n=14) during ACA DREADD self-administration experiment during each mating session. SA: self-administration; ML: mount latency (seconds); IL: intromission latency (seconds); EL: ejaculation latency (seconds); Cop. eff.: copulation efficiency (percentage). Ejaculation latency is not calculated on Perfusion Day as 10 minutes was not sufficient time for all animals to reach ejaculation (7/32 males showed ejaculation in all three groups and pERK expression did not correlate with display of ejaculation).

| **Figure #** | **Measure** | **Statistical Test** | **Factor** | **F value/t value** | **Main effect** | **Pairwise Comparison(s)** | **Pairwise effects** |
| --- | --- | --- | --- | --- | --- | --- | --- |
| 3D | Active Resp | Student’s t-test | -- | t(14) = 1.70, 2.0, 2.0 (one-tailed) | -- | veh C vs veh NC | Session 1: P = 0.045; Session 2: 0.020 |
|  |  |  |  |  |  | CNO C vs veh NC | Session 2: P = 0.025 |
| 3E | Active Resp | 2-way ANOVA | group | F(2,46) = 7.3 | P = 0.002 | within Cue R, veh C vs veh NC | P < 0.001 |
|  |  |  |  |  |  | within Cue R, veh C vs CNO C | P < 0.001 |
|  |  |  | session | F(1,46) = 64.1 | P < 0.001 | within veh NC, extinction vs Cue R | P = 0.009 |
|  |  |  |  |  |  | within veh C, extinction vs Cue R | P < 0.001 |
|  |  |  |  |  |  | within CNO C, extinction vs Cue R | P < 0.001 |
|  |  |  | group x session | F(2,46) = 6.1 | P = 0.004 | -- | -- |
| 3F | Active Resp | 2-way ANOVA | group | F(2,44) = 2.6 | P = 0.086 | No main effect therefore no post-hoc comparisons; group effect detected in Student’s t-test (next row) | -- |
|  |  | Student’s t-test | -- | t(12) = 2.40, 2.78 | -- | Within Meth R: veh NC vs veh C, CNO C vs veh C | P = 0.011, 0.004 |
|  |  | 2-way ANOVA | session | F(1,44) = 29.1 | P < 0.001 | within veh NC, extinction vs Meth R | P = 0.024 |
|  |  |  |  |  |  | within veh C, extinction vs Meth R | P < 0.001 |
|  |  |  |  |  |  | within CNO C, extinction vs Meth R | P = 0.006 |

**Table S6.** Detailed statistical analysis information for all results reported in main text for Figure 3. Vehicle non-concurrent (n=6), vehicle concurrent (n=12) and CNO concurrent (n=13).

| Group | # CaMKII cells  per mm^2^ | # pERK cells  per mm^2^ | # dual pERK + CaMKII | % pERK cells  expressing CaMKII | # cFos cells  per mm^2^ | # dual cFos + CaMKII | % cFos cells  expressing CaMKII |
| --- | --- | --- | --- | --- | --- | --- | --- |
| veh NC | 268.8 ± 20.3 | 150.0 ± 20.3 | 81.5 ± 10.5 | 54.8% ± 1.0% | 105.3 ± 15.9 | 7.2 ± 1.7 | 7.6% ± 2.2% |
| veh C | 268.0 ± 20.2 | 145.2 ± 11.8 | 80.2 ± 13.3 | 57.0 % ± 4.5% | 142.7 ± 16.7 | 6.5 ± 1.7 | 5.7 % ± 1.7% |
| CNO C | 271.3 ± 18.3 | 87.0 ± 18.1* | 50.7 ± 12.1* | 58.1% ± 2.6% | 247.2 ± 43.9* | 16.9 ± 5.1* | 6.9% ± 1.8% |

**Table S7.** Mean ± SEM numbers of cells immunoreactive for CaMKII, mating-induced pERK, or Meth-induced cFos, within the area containing the viral vector injection site (ACA) and expressed per square mm, in the 3 groups (veh NC n=6, veh C n=5, CNO C n=6; second cohort only) after completion of DREADD self-administration. *indicates significant difference versus both vehicle control groups (p<0.001-0.031).

| **Figure #** | **Measure** | **Statistical Test** | **Factor** | **F value/t value** | **Main effect** | **Pairwise Comparison(s)** | **Pairwise effects** |
| --- | --- | --- | --- | --- | --- | --- | --- |
| 4F | Distance traveled (cm) | 2-way RM ANOVA | group | F(3,21) = 8.8 | P < 0.001 | all CNO doses, Meth vs saline | P = 0.0045 - 0.005 |
| 4J | Active Resp | 2-way RM ANOVA | group | F(1,26) = 5.2 | P = 0.038 | C combined vs veh NC | Session 1: P = 0.001; Session 2: P = 0.011 |
| 4K | Active Resp | 2-way ANOVA | session | F(1,26) = 11.9 | P = 0.002 | within veh C, extinction vs Cue R | P = 0.006 |
|  |  |  |  |  |  | within CNO C, extinction vs Cue R | P = 0.0185 |

**Table S8.** Detailed statistical analysis information for all results reported for Figure 4. Panel F-G: veh sal (n=3), veh Meth (n=3), CNO sal (n=3), CNO Meth (n=3); Panels J-K vehicle non-concurrent (n=6), vehicle concurrent (n=6) and CNO concurrent (n=4).

| Group | # CaMKII cells  per mm^2^ | # pERK cells  per mm^2^ | # cFos cells  per mm^2^ | % pERKs cells  expressing CaMKII | % cFos cells  expressing CaMKII |
| --- | --- | --- | --- | --- | --- |
| veh NC | 342.6 ± 18 | 155.6 ± 15.5 | 118.5 ± 14.8 | 12.2 ± 3.2 | 9.7 ± 2.3 |
| veh C | 398.2 ± 52.1 | 157.4 ± 21.0 | 92.6 ± 12.4 | 8.0 ± 3.9 | 17.0 ± 4.5 |
| CNO C | 352.8 ± 80.8 | 102.8 ± 17.2* | 83.3 ± 18.4 | 0.2 ± 0.1 | 0.1 ± 0 |

**Table S9.** Mean ± SEM numbers of cells immunoreactive for CaMKII, mating-induced pERK, or Meth-induced cFos, within the area containing the viral vector injection site (vmPFC) and expressed per square mm, in the 3 groups (veh NC n=6, veh C n=6, CNO C n=4) after completion of DREADD self-administration. *indicates significant difference versus both vehicle control groups (p=0.028 and 0.05).

|  |  |  |
| --- | --- | --- |
|  |  |  |
|  | **TOTAL INFUSIONS EARNED** | **TOTAL METH INTAKE (mg/kg)** |
| Veh concurrent | 91.5 ± 6.3 | 3.7 ± 0.258 |
| Veh non-concurrent | 113.8 ± 7.3 | 4.6 ± 0.295 |
| CNO concurrent | 113.5 ± 7.6 | 4.6 ± 0.296 |
|  |  |  |

**Table S10.** Average Meth intake across drug self-administration and chemogenetic inactivation of vmPFC; veh: vehicle, CNO: clozapine-*N*-oxide, Meth: methamphetamine. No significant differences were detected between groups. All data are expressed as Mean ± SEM.
